# An Insulin‐Exosome‐TNFAIP8 Axis Drives Stromal Fibrosis and Therapeutic Resistance in Pancreatic Cancer

**DOI:** 10.1002/advs.202515606

**Published:** 2026-02-19

**Authors:** Zhenyu Li, Li Chen, Tao Wang, Haiyang Jiang, Huijuan Wang, Mengyu Li, Guanpeng Xie, Chunhua Xi, Han Yan, Chunhui Lu, Chenchen Li, Hanyu Zhu, Feihu Sun, Lingdi Yin, Jun Yu, Yi Miao

**Affiliations:** ^1^ Pancreas Center The Affiliated BenQ Hospital of Nanjing Medical University Nanjing Jiangsu Province China; ^2^ Pancreas Center National Clinical Research Center for Cancer Tianjin Medical University Cancer Institute & Hospital Tianjin China; ^3^ Pancreas Center The First Affiliated Hospital of Nanjing Medical University Nanjing Jiangsu Province China

**Keywords:** fibrosis, pancreatic ductal adenocarcinoma, STAT1, TNFAIP8, ubiquitination

## Abstract

Hyperinsulinemia, a hallmark of obesity and type 2 diabetes, is an emerging risk factor for pancreatic ductal adenocarcinoma (PDAC), yet its contribution to tumor progression and stromal remodeling remains unclear. Here, we identify an insulin–exosome–TNFAIP8–STAT1 signaling axis that is associated with fibroblast phenotypic remodeling and desmoplastic progression. Insulin activates PI3K/AKT‐RAB3A signaling to enhance secretion of TNFAIP8‐enriched exosomes from PDAC cells. Internalized TNFAIP8 recruits the E3 ligase TRIM21 to facilitate STAT1 ubiquitination and degradation, leading to the induction of myofibroblastic CAF–associated features, accompanied by enhanced extracellular matrix deposition and tumor growth. High TNFAIP8 expression in patient tumors correlates with fibrosis and poor prognosis. In orthotopic models, TNFAIP8 silencing or lipid nanoparticle‐mediated shTNFAIP8 delivery reduced fibrosis, suppressed tumor progression, and enhanced gemcitabine efficacy without evident toxicity, suggesting the feasibility of a therapeutic approach. These findings uncover a mechanistic framework linking metabolic dysregulation to fibroinflammatory remodeling in PDAC, and nominate TNFAIP8 as a promising stromal‐targeted therapeutic candidate.

## Graphical abstract

In pancreatic ductal adenocarcinoma (PDAC), insulin is associated with activation of PI3K/AKT–RAB3A signaling and enhanced secretion of TNFAIP8‐enriched exosomes from tumor cells. Uptake of these exosomes by fibroblasts is linked to TRIM21‐dependent STAT1 degradation and the emergence of myofibroblastic CAF–associated features, accompanied by increased fibrotic remodeling and tumor growth. These findings suggest that targeting TNFAIP8 may represent a feasible stromal‐directed strategy to modulate hyperinsulinemia‐associated tumor progression in PDAC.

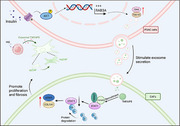

## 1 Introduction

Pancreatic ductal adenocarcinoma (PDAC) remains one of the deadliest malignancies, with a 5‐year survival rate of only 13% [1]. Beyond its intrinsically aggressive biology, PDAC is marked by an exceptionally dense and heterogeneous desmoplastic stroma that profoundly drives therapeutic resistance [2, 3, 4]. Cancer‐associated fibroblasts (CAFs) constitute the dominant stromal population, and single‐cell analyses have identified multiple functionally distinct CAF subsets—including myofibroblastic CAFs (myCAFs), inflammatory CAFs (iCAFs), and antigen‐presenting CAFs (apCAFs). These subsets exert divergent influences on tumor progression, immune evasion, and therapeutic response. Importantly, dynamic interconversion among CAF states contributes to fibrosis severity and treatment failure; however, the upstream tumor‐derived signals governing CAF subtype specification remain poorly defined [5, 6].

Hyperinsulinemia, a hallmark of type 2 diabetes and exogenous insulin therapy, is epidemiologically and mechanistically linked to PDAC risk and progression [7, 8, 9, 10, 11, 12]. Notably, insulin secreted by pancreatic β cells enters the portal circulation and exposes the pancreas to locally elevated insulin concentrations. Our previous work showed that insulin enhances invasion and migration of KRAS‐mutant pancreatic ductal cells by upregulating MMP‐2 [13], and Zhang et al. similarly reported that excessive insulin signaling accelerates acinar injury and inflammation [14]. At the tumor‐cell level, insulin promotes proliferation and survival through INSR‐mediated PI3K/AKT and MAPK pathways [14, 15, 16, 17, 18]. Physiological insulin levels (0.2–20 nmol/L) have been reported to protect pancreatic islet cells through insulin receptor activation, suggesting that pancreatic tissues—and potentially stromal elements—exist within an insulin‐enriched microenvironment. Yet whether such heightened insulin signaling directly modulates stromal remodeling, particularly CAF subtype transitions, remains unknown. Furthermore, Tumor‐derived exosomes are key mediators of fibroblast education [19, 20], but the specific exosomal cargos that couple metabolic cues such as hyperinsulinemia to CAF fate determination have not been defined.

In addition, Tumor necrosis factor–α–induced protein 8 (TNFAIP8), the founding member of the TIPE family, has been implicated in regulating apoptosis, inflammation, and tumor progression. Aberrant TNFAIP8 expression has been reported in multiple malignancies, including pancreatic, lung, and breast cancers, where it promotes tumor growth, invasion, and metastasis through NF‐κB and Hippo pathway modulation [21]. Although TNFAIP8 has been linked to oncogenic signaling, its regulatory mechanisms—particularly those involving protein stability—remain incompletely understood. It is notable that the ubiquitin–proteasome system also plays a central role in shaping tumor behavior by controlling protein turnover. Dysregulated ubiquitination contributes to PDAC progression and chemoresistance, in part by remodeling the tumor microenvironment [22].

In this study, we identify insulin‐induced exosomal secretion of TNFAIP8 from PDAC cells as a previously unrecognized mechanism linking metabolic signaling to stromal remodeling. TNFAIP8‐enriched exosomes enhanced CAF proliferation and activation in a paracrine‐like manner. Mechanistically, TNFAIP8 interacted with STAT1 in CAFs and facilitated its K48‐linked ubiquitination through recruitment of the E3 ligase TRIM21, leading to STAT1 degradation and promoting myCAFs‐associated phenotypic features. In vivo, TNFAIP8 knockdown attenuated insulin‐driven tumor growth and stromal fibrosis. Moreover, lipid nanoparticle (LNP)–mediated delivery of shTNFAIP8 reduced tumor burden, mitigated stromal remodeling, and improved gemcitabine sensitivity. Collectively, these findings support an insulin–exosome–TNFAIP8 signaling axis that links metabolic cues to CAF phenotypic remodeling and suggest TNFAIP8 as a potential stromal‐targeted therapeutic candidate in PDAC.

## 2 Result

### 2.1 Insulin Drives CAF Proliferation and Fibrosis via Tumor‐Mediated Paracrine Signaling in PDAC

**FIGURE 1:**
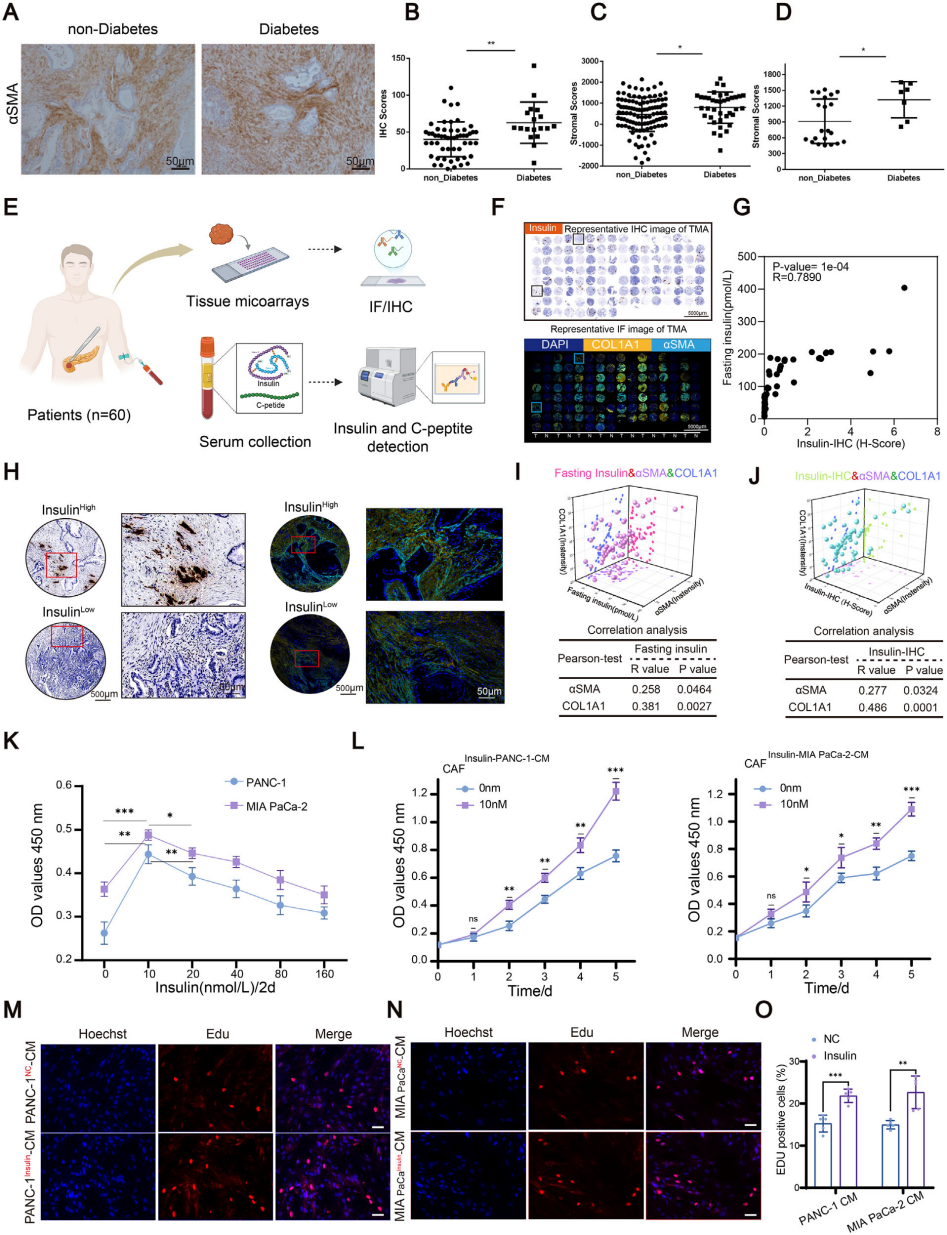
Insulin indirectly promotes fibrosis and CAF activation in PDAC. (A) Representative IHC staining of α‐SMA in PDAC tumor tissues from non‐diabetic (*n* = 54) and diabetic (*n* = 18) patients in our institutional cohort. (B) Quantification of α‐SMA IHC scores in diabetic versus non‐diabetic PDAC samples. (C) Stromal scores derived from TCGA PDAC cohort, diabetic (*n* = 38) compared to non‐diabetic (*n* = 109). (D) Single‐cell RNA‐seq analysis of Stromal scores in PDAC samples with or without diabetes (GSA: CRA001160), diabetic (*n* = 7) compared to non‐diabetic (*n* = 21). (E) Schematic illustration of the study design. Fasting serum samples and matched PDAC TMAs were collected from 60 patients. Serum insulin and C‐peptide levels were measured, and TMAs tissues were analyzed by IHC and multiplex IF. (F) Representative insulin IHC staining and multiplex IF images of TMAs showing DAPI (blue), COL1A1 (yellow), and αSMA (wathet). Scale bars, 5000 µm. (G) Positive correlation between fasting serum insulin levels and tumor insulin IHC H‐score. (H) Representative IHC and multiplex IF images from insulin‐high and insulin‐low PDAC tumors, showing differences in stromal distribution and extracellular matrix organization. Scale bars, 500 µm and 50 µm. (I) 3D quantification correlation analysis of fasting insulin levels with αSMA and COL1A1 fluorescence intensities. (J) 3D quantification correlation analysis of insulin IHC H‐score with αSMA and COL1A1 fluorescence intensities. (K) CCK‐8 assay performed to assess the proliferative response of PDAC tumor cells to varying concentrations of insulin (*n* = 3, biological replicates). (L) CCK‐8 assays to evaluate CAF proliferation after stimulation with conditioned medium (CM) from insulin‐treated tumor cells (PANC‐1, MIA PaCa‐2) (*n* = 3, biological replicates). (M–O) EdU staining and quantification for CAFs treated with CM from insulin‐pretreated tumor cells (*n* = 5, biological replicates). Data are presented as mean ± SD. Statistical comparisons between two groups were performed using a two‐tailed unpaired Student's *t*‐tests (B, C, D, K, L, O). Correlation analyses were conducted using Pearson's correlation coefficient (G, I, J). Significance thresholds are shown as: *p < 0.05, **p < 0.01, ***p < 0.001.

To investigate the link between hyperinsulinemia and stromal fibrosis in PDAC, we first assessed α‐SMA expression in tumor tissues in our institutional cohort and found significantly higher α‐SMA–positive stromal regions in diabetic patients (Figure 1A,B). This was supported by TCGA‐based stromal scores and single‐cell RNA‐seq data, which showed increased stromal scores and elevated activation of CAF‐associated fibrotic programs in diabetic PDAC samples (Figure 1C,D).

Notably, hyperinsulinemia, a defining feature of type 2 diabetes and exogenous insulin therapy, was associated with increased stromal activation, to directly assess the role of insulin, we collected paired fasting serum samples and tissue microarray from 60 PDAC patients (Figure 1E). Serum insulin and C‐peptide levels were quantified, and matched tumor tissues were analyzed by IHC and multiplex immunofluorescence (Figure 1F). IHC and IF staining on the PDAC tissue microarray further validated enhanced stromal activation, assessed through the ECM/fibrosis markers αSMA and COL1A1. Fasting serum insulin levels positively correlated with tumor insulin IHC H‐scores, validating tissue insulin staining as a reflection of local insulin exposure. Multiplex IF analysis revealed that insulin‐high tumors exhibited increased α‐SMA–positive stromal regions and enhanced collagen deposition compared with insulin‐low tumors. 3D quantification correlation analyses further demonstrated positive associations between fasting insulin levels or insulin IHC scores and α‐SMA and COL1A1 fluorescence intensities (Figure 1I,J), indicating a strong link between insulin exposure and fibrotic stromal remodeling. In contrast, serum C‐peptide levels showed no significant correlation with tumor insulin IHC scores and were not associated with stromal fibrosis, as reflected by α‐SMA and COL1A1 expression (Figure S1A–C), This is consistent with the limitation that C‐peptide reflects endogenous insulin secretion and does not account for exogenous insulin administration in treated patients.

To investigate the role of hyperinsulinemia in the PDAC tumor microenvironment, we first isolated and validated primary CAFs from human PDAC tissues and then assessed INSR expression in human PDAC specimens (Figure S1D–F). Multiplex IF analysis revealed that INSR‐high tumors exhibited significantly enhanced stromal activation, as indicated by elevated α‐SMA levels, compared with INSR‐low tumors (Figure S1G). INSR expression was detected in both CK19^+^ tumor epithelial cells and α‐SMA^+^ CAFs, indicating that insulin signaling is potentially active across multiple cellular compartments within the tumor microenvironment. Consistently, immunoblotting identified PDAC cell lines and CAFs with relatively high INSR expression. Based on these findings, we selected INSR‐high tumor cells (PANC‐1, MIA PaCa‐2) and CAFs (CAF3, CAF4) as experimental models to further dissect the contribution of hyperinsulinemia to tumor–stroma interactions in PDAC (Figure S1H,I). Insulin promoted tumor cell proliferation but had no direct effect on CAFs (Figure S1K–O; Figure 1K), consistent with the possible involvement of a paracrine mechanism and in a two‐step CM model (Figure S1J), CM from insulin‐treated tumor cells significantly enhanced CAF proliferation (Figure 1L–O). These findings suggest that insulin may facilitate CAF proliferation and stromal fibrotic responses through tumor‐mediated paracrine signaling in PDAC.

### 2.2 Insulin Enhances Tumor‐Derived Exosome Release and is Associated With Increased CAF Proliferation

**FIGURE 2:**
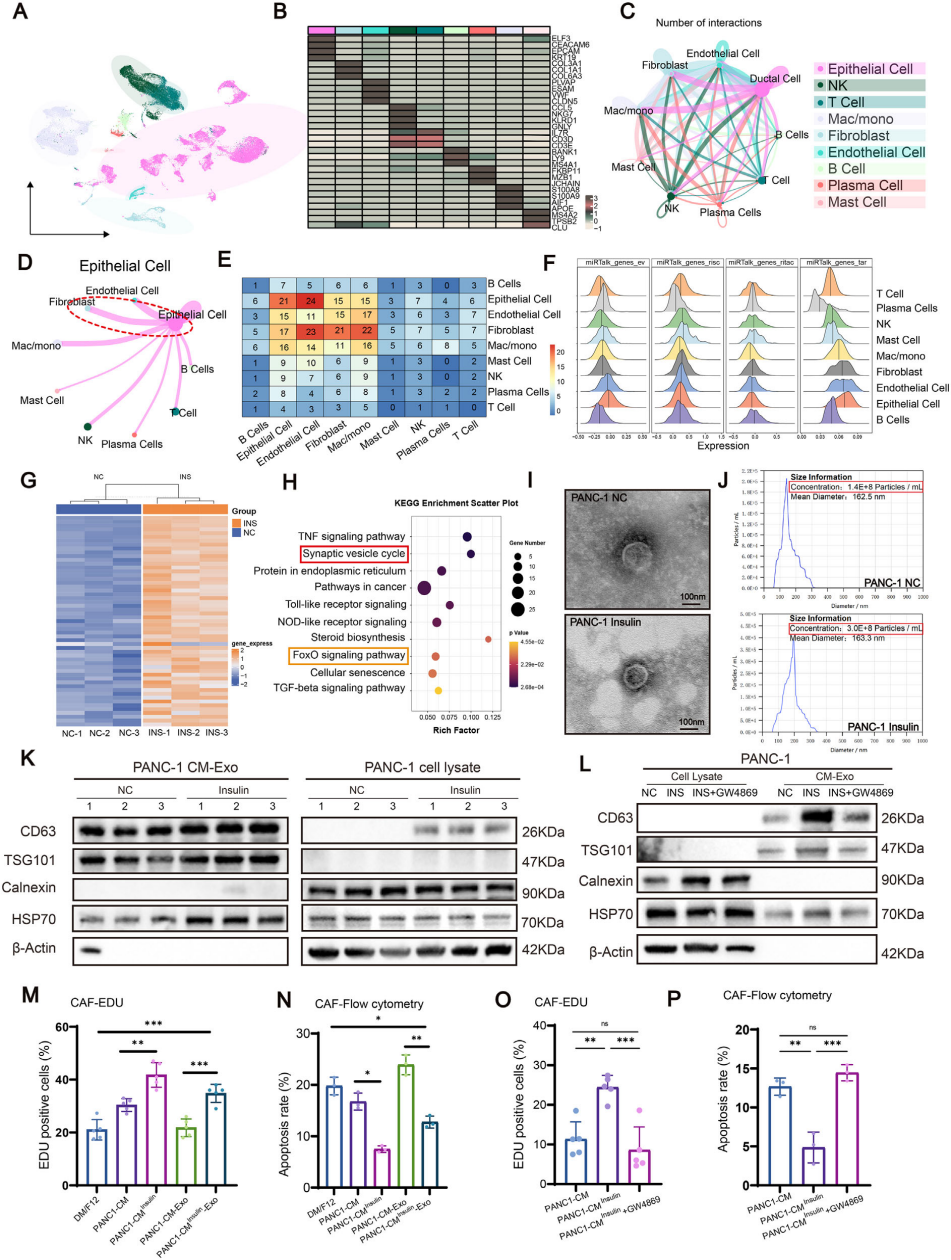
Insulin‐driven exosome release from PDAC cells modulates CAF proliferation and apoptosis. (A) UMAP projection of single‐cell RNA‐sequencing data from the PDAC dataset (GSE205013), showing major cell populations within the tumor microenvironment. (B) Heatmap of representative marker genes used for cell‐type annotation, identifying epithelial cells, fibroblasts, endothelial cells, and immune subsets including T cells, NK cells, B cells, plasma cells, macrophages/monocytes, and mast cells. (C) Global cell–cell interaction network inferred from secreted protein–mediated communication (CDE analysis). Nodes represent cell types, and edges indicate the number of predicted secretory interactions between cell populations. (D) Epithelial cell–centered interaction network highlighting directional secretory signaling from tumor epithelial cells to stromal and immune compartments, with prominent connections to fibroblasts. (E) Heatmap showing the number of secreted protein–mediated interactions between different cell types, illustrating the overall communication landscape. (F) Ridge plots showing the distribution of expression scores for miRTalk‐predicted gene sets across major cell types, including extracellular vesicle–associated genes (miRTalk_genes_ev), risk‐associated genes (miRTalk_genes_risc), receptor–interaction–associated genes (miRTalk_genes_ritac), and target genes (miRTalk_genes_tar). These analyses illustrate cell‐type–specific patterns of miRNA‐related regulatory and secretory gene activity within the PDAC tumor microenvironment. (G) Heatmap showing the top differentially expressed genes in MIA CaPa‐2 cells treated with or without insulin, as identified by RNA‐seq. (H) KEGG pathway enrichment analysis of differentially expressed genes from RNA‐seq data. (I) Transmission electron microscopy (TEM) images of exosomes isolated from PANC‐1 conditioned media with or without insulin stimulation. Scale bars, 100 nm. (J) Nanoparticle tracking analysis (NTA) of exosome size distribution and concentration from control and insulin‐treated PANC‐1 cells. (K) Western blot validation of exosomal markers (CD63, TSG101) and negative control (Calnexin) and HSP70 in exosome fractions and corresponding cell lysates from PANC‐1 cells treated with or without insulin. (L) Western blot analysis confirming the effect of the exosome inhibitor GW4869 on exosomal protein markers in PANC‐1 cells and conditioned media. (M, N) Proliferation (M, EdU incorporation, *n* = 5 biological replicates) and apoptosis (N, flow cytometry, *n* = 3 biological replicates) of CAFs cultured with tumor cell–derived conditioned medium or isolated exosomes, as indicated. (O, P) Proliferation (O, EdU incorporation, *n* = 5 biological replicates) and apoptosis (P, flow cytometry, *n* = 3 biological replicates) of CAFs following insulin stimulation in the presence or absence of the exosome secretion inhibitor GW4869. Data are presented as mean ± SD and for multiple‐group comparisons, statistical significance was assessed using one‐way ANOVA followed by Tukey's post hoc test (M, N, O, P). Significance thresholds: *p < 0.05, **p < 0.01, ***p < 0.001.

To delineate cell–cell communication networks within the PDAC tumor microenvironment, we analyzed a published single‐cell RNA‐sequencing dataset (GSE205013). UMAP projection revealed distinct clustering of major cellular compartments, including epithelial cells, fibroblasts, endothelial cells, and multiple immune subsets (Figure 2A). Cell‐type annotation was confirmed by canonical marker gene expression (Figure 2B), establishing a reliable framework for downstream intercellular communication analyses. We next inferred global secreted protein–mediated cell–cell interactions using CDE analysis. The resulting interaction network demonstrated extensive communication among epithelial, fibroblast, endothelial, and immune populations, with fibroblasts emerging as one of the most highly connected stromal cell types (Figure 2C). An epithelial cell–centered interaction network further highlighted strong directional signaling from tumor epithelial cells toward fibroblasts, suggesting epithelial cells as a dominant source of stromal‐modulating secretory cues (Figure 2D). Quantitative interaction matrices confirmed that epithelial–fibroblast communication ranked among the most prominent secretory interactions within the tumor microenvironment (Figure 2E). In parallel, analysis of miRTalk‐predicted gene sets revealed distinct cell‐type–specific expression patterns. Genes associated with extracellular vesicles, receptor–interaction modules, and miRNA‐regulated targets were enriched in epithelial cells and fibroblasts, supporting a potential role for vesicle‐mediated and post‐transcriptional regulatory communication between these compartments (Figure 2F).

To complement secreted protein–based interactions, we further evaluated cytokine and growth factor ligand–receptor signaling using an independent inference framework. This analysis similarly demonstrated dense intercellular communication across cell types, with fibroblasts and epithelial cells forming a major bidirectional signaling axis (Figure S2A). Epithelial‐centered ligand–receptor networks again underscored fibroblasts as a principal recipient of tumor‐derived signaling inputs (Figure S2B). These complementary analyses consistently identify tumor epithelial cells and fibroblasts as key communicative hubs within the PDAC microenvironment, supporting a model in which epithelial‐derived secretory programs may play a central role in shaping CAF activation states.

To investigate how insulin stimulation of tumor cells indirectly modulates CAF behavior, we performed RNA‐seq in MIA CaPa‐2 cells with or without insulin stimulation, which revealed significant changes in gene expression related to vesicle transport pathways (Figure 2G,H). Transmission electron microscopy (TEM) and nanoparticle tracking analysis (NTA) confirmed that insulin treatment increased the quantity of exosomes released by both PANC‐1 and MIA PaCa‐2 cells without significantly altering their size (Figure 2I,J; Figure S3A,B). Western blot analysis validated the enrichment of exosomal markers (CD63, TSG101) in exosome fractions and showed that the exosome secretion inhibitor GW4869 effectively reduced these markers in conditioned media (CM) (Figure 2K,L; Figure S3C,D).

To assess the functional consequence of increased tumor‐derived exosomes on CAF behavior, we treated CAFs with CM or isolated exosomes from control and insulin‐treated pancreatic cancer cells. EdU staining and flow cytometry demonstrated that insulin‐induced exosomes significantly promoted CAF proliferation and decreased cell apoptosis, whereas pharmacological inhibition of exosome release with GW4869 markedly attenuated CAF proliferation and increased apoptotic cell fractions. (Figure 2M–P; Figures S2C–F and S3E–H). Together, these results suggest that insulin enhances exosome secretion from pancreatic cancer cells, which in turn modulates CAF proliferation, thereby potentially contributing to stromal remodeling in the tumor microenvironment.

### 2.3 Insulin Activates PI3K/AKT–RAB3A Signaling, Which Is Associated With Enhanced Exosome Release and Increased Stromal Fibrosis in PDAC in Vitro and In Vivo

**FIGURE 3:**
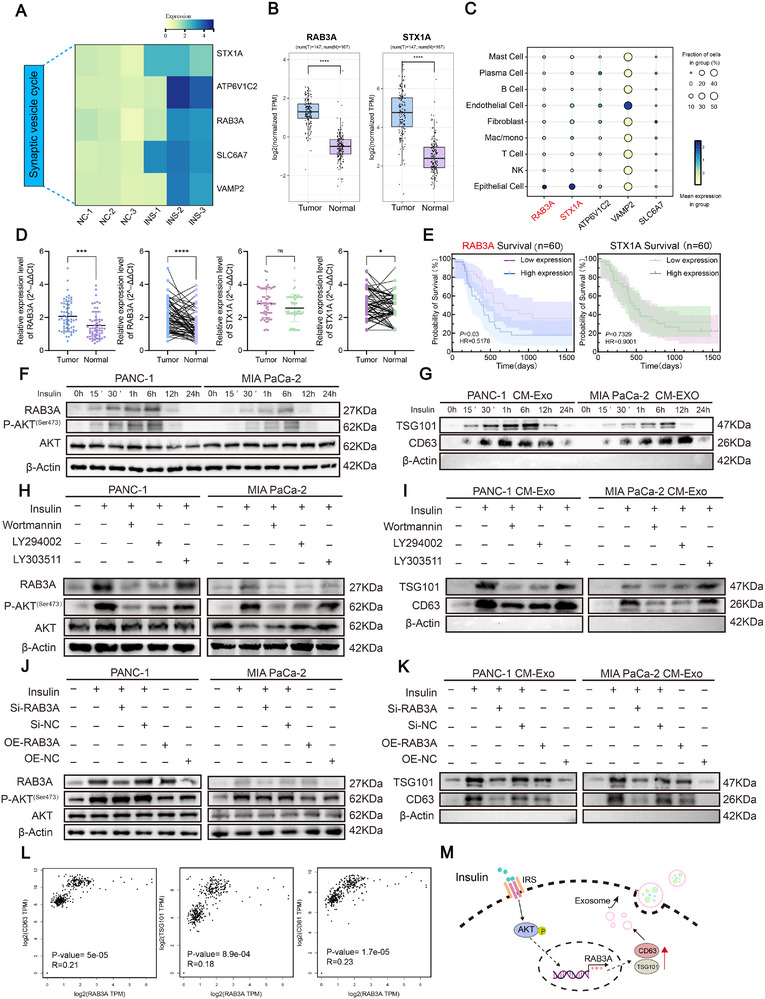
Insulin upregulates RAB3A via PI3K/AKT signaling to promote exosome release in PDAC cells (A) Heatmap showing the expression patterns of synaptic vesicle cycle–associated genes under control (NC) and insulin‐treated (INS) conditions. (B) Differential expression of RAB3A and STX1A between pancreatic ductal adenocarcinoma (PDAC) and adjacent normal tissues based on normalized TCGA‐PDAC transcriptomic data (log2 TPM). (C) Dot plot illustrating the cell‐type–specific expression of synaptic vesicle cycle–related genes in PDAC single‐cell RNA‐seq data (GSE205013). Dot size represents the proportion of expressing cells, and color intensity indicates mean expression levels. (D) qRT‐PCR analysis of RAB3A and STX1A mRNA levels in PDAC tumor and adjacent normal tissues (*n* = 60), presented as scatter and paired line plots (2^–ΔΔCt^). (E) Kaplan–Meier curves of overall survival in PDAC patients (*n* = 60) stratified by RAB3A (left) and STX1A (right) expression. (F) Time‐course Western blot analysis of RAB3A and P‐AKT ^(Ser473)^ expression in PANC‐1 and MIA PaCa‐2 cells treated with insulin. (G) Western blot showing time‐course expression of exosomal markers (TSG101, CD63) in CM‐derived exosomes from insulin‐treated cells. (H) Western blot validating effects of PI3K/AKT inhibitors (Wortmannin, LY294002, LY303511) on insulin‐induced P‐AKT ^(Ser473)^ and RAB3A levels. (I) Western blot validating effects of PI3K/AKT inhibitors on exosomal marker expression in CM‐derived exosomes. (J) Western blot analysis of P‐AKT ^(Ser473)^ and RAB3A levels after RAB3A knockdown (Si‐RAB3A) or overexpression (OE‐RAB3A) in PANC‐1 and MIA PaCa‐2 cells. (K) Western blot of exosomal markers in CM‐derived exosomes from cells with RAB3A knockdown or overexpression. (L) TCGA correlation analysis showing RAB3A expression is positively correlated with CD63, TSG101, and CD81 levels in PDAC samples. (M) Schematic model showing that insulin activates PI3K/AKT to upregulate RAB3A and promote exosome release in PDAC cells. Data are presented as mean ± SD. For comparisons between tumor versus normal tissues (B, D), two‐tailed paired Student's *t*‐test was used. Survival differences were assessed using the log‐rank (Mantel–Cox) test (E). Significance thresholds: *p < 0.05, **p < 0.01, ***p < 0.001.

RNA‐seq analysis revealed that insulin stimulation enriched vesicle trafficking pathways, with upregulation of core synaptic vesicle cycle genes (STX1A, ATP6V1C2, RAB3A, SLC6A7, VAMP2) in PDAC cells (Figure 3A). which was further supported by TCGA analysis showing higher expression of RAB3A, STX1A in pancreatic tumor tissues than in normal tissues (Figure 3B; Figure S4A). Single‐cell analysis revealed that vesicle trafficking genes, particularly RAB3A and STX1A, were highly enriched in tumor epithelial cells but scarcely expressed in stromal or immune cells (Figure 3C). This indicates that insulin‐regulated vesicle/exosome programs are primarily tumor cell–intrinsic. qRT‐PCR showed elevated RAB3A expression in PDAC tumors, while STX1A remained largely unchanged (Figure 3D). Kaplan–Meier analysis indicated that high RAB3A expression was associated with poorer survival, whereas STX1A showed no prognostic significance (Figure 3E).

Time‐course Western blotting with densitometric quantification demonstrated that insulin induced a sustained, time‐dependent increase in P‐AKT (Ser473) and RAB3A protein levels in both PANC‐1 and MIA PaCa‐2 cells (Figure 3F and Figure S4B). Concurrently, insulin enhanced exosomal marker (TSG101 and CD63) levels in cell‐derived exosomes (Figure 3G; Figure S4C). Treatment with PI3K/AKT pathway inhibitors (Wortmannin, LY294002, LY303511) markedly suppressed insulin‐induced RAB3A upregulation and AKT activation (Figure 3H; Figure S4D) and reduced exosome marker levels in the cell‐derived exosomes (Figure 3I and Figure S4E). Knockdown or overexpression of RAB3A did not alter AKT phosphorylation, but markedly affected exosome secretion (Figure 3J,K; Figure S5A,B). TCGA correlation analysis demonstrated that RAB3A expression positively correlated with exosomal marker genes CD63, TSG101, and CD81 in PDAC samples (Figure 3L).

To validate these findings in vivo, we established both subcutaneous and orthotopic xenograft models using PANC‐1 and MIA PaCa‐2 cells with CAF co‐injection. RAB3A was efficiently overexpressed in PANC‐1 and MIA PaCa‐2 cells, as confirmed at both mRNA and protein levels (Figure S5C,D). RAB3A overexpression significantly promoted tumor growth in insulin‐treated mice, whereas administration of the exosome secretion inhibitor GW4869 suppressed this effect (Figure S5E–G). Similarly, orthotopic models confirmed that insulin‐driven tumor progression was dependent on RAB3A‐mediated exosome release, which was attenuated by GW4869 (Figure S5G–I). Quantification of tumor fluorescence intensity further supported these results (Figure S5J). IHC analysis of tumor tissues showed that RAB3A overexpression markedly increased the expression of fibrosis markers (COL1A1 and α‐SMA) and the proliferation marker Ki67, whereas inhibition of exosome release with GW4869 largely attenuated these effects (Figure S6A–B).

In conclusion, these results show that insulin stimulation is accompanied by activation of PI3K/AKT signaling, increased RAB3A expression, and enhanced exosome secretion in PDAC cells. These alterations correlate with stromal activation, and inhibition of exosome release mitigated several of these effects. A working model summarizing this regulatory axis is presented in Figure 3M.

### 2.4 TNFAIP8‐Enriched Exosomes Modulate CAF Activation in PDAC

**FIGURE 4:**
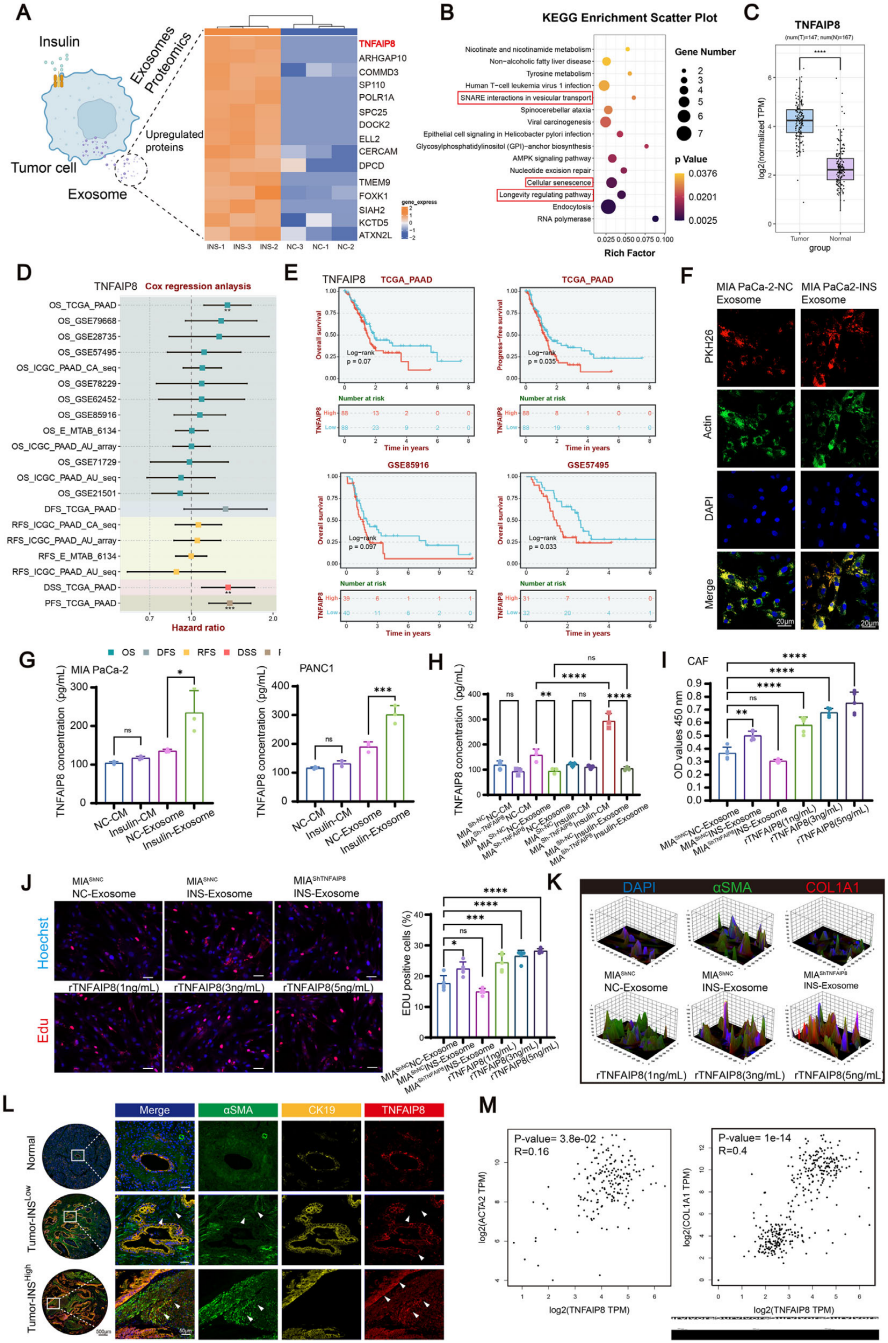
TNFAIP8 is enriched in insulin‐stimulated PDAC exosomes and promotes CAF activation. (A) Heatmap of differentially expressed proteins in exosomes from insulin‐treated versus control MIA PaCa‐2 cells. (B) KEGG pathway enrichment analysis of upregulated exosomal proteins. (C) Expression of TNFAIP8 based on normalized TCGA‐PAAD data. (D) Forest plot summarizing Cox regression analyses linking TNFAIP8 expression to survival outcomes across multiple datasets (TCGA, GSE79668, GSE28735, GSE57495, ICGC, GSE62452, GSE85916, MTAB_6134, GSE71729, GSE21501). (E) Kaplan‐Meier survival analyses for TNFAIP8 expression in PDAC cohorts. (TCGA, GSE85916, GSE57495). Data were analyzed using the BEST (Biomarker Evaluation & Survival Tool) database (D, E). (F) IF images showing uptake of exosomes by CAFs. Scale bars, 20 µm. (G) ELISA quantification of TNFAIP8 levels in cell supernatants and exosomes from indicated PDAC cells (MIAPaCa‐2, PANC1) (*n* = 3 biological replicates). (H) ELISA measurement of TNFAIP8 concentration in CM and exosome fractions collected from MIA PaCa‐2 cells under control or insulin treatment, with sh‐NC or sh‐TNFAIP8 expression. (*n* = 3 biological replicates). (I) CCK‐8 assay for CAF viability after exosome or rTNFAIP8 treatment (*n* = 3 biological replicates). (J) Representative EdU staining images and quantification of CAF proliferation under indicated treatments (*n* = 5 biological replicates). Scale bars, 50 µm. (K) 3D fluorescence intensity plots showing α‐SMA and COL1A1 expression in CAFs under indicated treatments. (L) Multiplex IF staining of human PDAC tissues showing co‐localization of TNFAIP8 with α‐SMA and CK19, Samples include normal pancreas, Tumor‐INS‐Low, and Tumor‐INS‐High groups. The Tumor‐INS‐High group consists of PDAC patients with elevated insulin levels in both tumor tissue and matched fasting serum, whereas Tumor‐INS‐Low represents patients with lower insulin levels. (TMA, *n* = 60; Tumor‐INS‐High, *n* = 15; Tumor‐INS‐Low, *n* = 45). Scale bars: 500 and 50 µm. (M) Correlation analyses between TNFAIP8 expression and stromal markers (ACTA2 and COL1A1) in TCGA PDAC samples. Statistical comparisons between two groups were performed using a two‐tailed unpaired Student's *t*‐tests (C). Data in (G, H, I, J) are shown as mean ± SD, analyzed by one‐way ANOVA with Tukey's test; Significance thresholds: ns, not significant; *p < 0.05; **p < 0.01; ***p < 0.001; ****p < 0.0001.

To further investigate downstream targets of insulin‐induced exosome release, we performed proteomic analysis of exosomes from insulin‐treated MIA PaCa‐2 cells, identifying TNFAIP8 as a notably upregulated protein (Figure 4A). KEGG analysis showed these exosomal proteins were enriched in pathways related to vesicle transport and tumor progression (Figure 4B). TCGA data confirmed higher TNFAIP8 mRNA levels in PDAC tissues versus normal (Figure 4C), with survival analyses linking high TNFAIP8 to poor prognosis across cohorts (Figure 4D–E). IF staining demonstrated effective uptake of exosomes by CAFs (Figure 4F). ELISA showed elevated TNFAIP8 in exosomes from insulin‐treated PDAC cells (Figure 4G). ELISA analysis showed that RAB3A knockdown or overexpression had little effect on TNFAIP8 levels in bulk conditioned medium but markedly altered TNFAIP8 enrichment in tumor cell–derived exosomes from both PANC‐1 and MIA PaCa‐2 cells (Figure S7A–B). Efficient TNFAIP8 silencing was confirmed by qRT–PCR and immunoblotting (Figure S7C–D). Under insulin stimulation, exosomal TNFAIP8 secretion was significantly increased, whereas TNFAIP8 knockdown selectively abolished this effect without altering non‐exosomal TNFAIP8 levels (Figure 4H), indicating that insulin–RAB3A signaling preferentially regulates exosomal TNFAIP8 release rather than total expression. Functionally, insulin‐induced exosomes and rTNFAIP8 increased CAF viability and proliferation (Figure 4I,J). 3D‐Multiplex IF analyses demonstrated higher α‐SMA and COL1A1 levels in CAFs treated with TNFAIP8‐rich exosomes or recombinant protein, suggesting TNFAIP8 may contribute to CAF activation and fibrotic remodeling (Figure 4K; Figure S7E).

In PDAC TMA, multiplex IF staining revealed co‐localization of TNFAIP8 with CK19, with particularly high expression in tumor stroma from Tumor‐INS‐High patients (Figure 4L; Figure S7F,G). Analysis of clinicopathological features revealed that high TNFAIP8 expression was linked to advanced TNM stage and enhanced stromal activation, as reflected by increased α‐SMA fluorescence intensity. Similar patterns were observed in cases with concurrent TNFAIP8 and α‐SMA expression, in addition, fasting serum insulin levels were positively associated with TNFAIP8‐associated stromal activation in PDAC (Table 1). IF analysis of orthotopic pancreatic tumors in nude mice showed that insulin stimulation and RAB3A overexpression were associated with increased αSMA^+^ stromal activation and elevated RAB3A and TNFAIP8 signals in tumors co‐implanted with CAFs, whereas inhibition of exosome release by GW4869 attenuated these effects (Figure S7H,I). These observations in vivo are consistent with a role for insulin–RAB3A–dependent exosomal signaling in modulating CAF activation within the tumor microenvironment. TCGA analysis further confirmed positive correlations between TNFAIP8 and stromal markers ACTA2 and COL1A1 (Figure 4M). These results indicate that insulin‐induced TNFAIP8 is selectively enriched in PDAC‐derived exosomes and may contribute to CAF activation and stromal remodeling.

**TABLE 1:** Correlation Between TNFAIP8 Expression and Clinicopathological Characteristics in PDAC Patients (*n* = 60).

Characteristics	N of cases	TNFAIP8+level			TNFAIP8+αSMA+ level		
		High	Low	p‐value	High	Low	*p*‐value
Total cases	60	23	37		27	33	
Age				1.000			0.939
≥50	52	20	32		24	28	
<50	8	3	5		3	5	
Gender				0.146			0.466
Male	32	15	17		13	19	
Female	28	8	20		14	14	
T stage				0.776			0.176
T1‐2	43	16	27		17	26	
T3‐4	17	7	10		10	7	
N stage				0.180			0.118
N0	22	6	16		7	15	
N1‐N3	38	17	21		20	18	
TNM stage(%)							
I‐II	41	12	29	0.034	14	27	0.013
III	19	11	8		13	6	
Preoperative CA199 KU/L				0.515			0.072
≤37	13	6	7		3	10	
38‐1000	35	14	21		20	15	
≥1000	12	3	9		4	8	
Fasting insulin pmol/L				0.0028			0.0336
≤17.8	3	1	2		1	2	
17.8‐173.0	42	11	32		16	26	
≥173.0	15	11	3		10	5	
αSMA+ Fluorescence intensity		27.5(24.9,32.5)	23.0 (21.8,25.6)	0.003	26.5(24.9,32.5)	23.0(21.8,24.9)	0.002

### 2.5 TNFAIP8 Facilitates STAT1 Degradation Through K48‐Linked Ubiquitination

**FIGURE 5:**
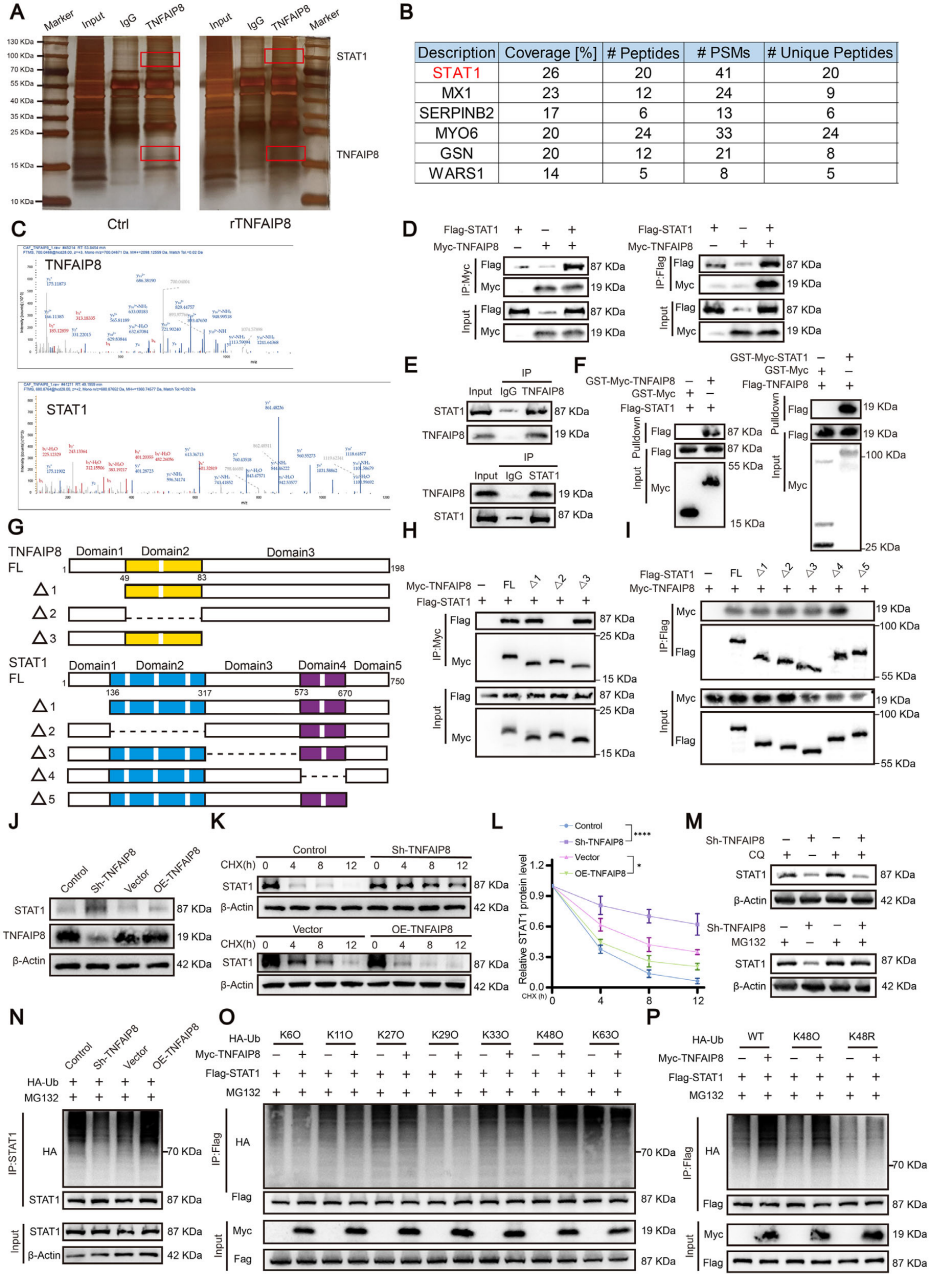
TNFAIP8 interacts with STAT1 and promotes its proteasomal degradation through K48‐linked ubiquitination. (A) Silver staining of co‐immunoprecipitation (Co‐IP) samples using anti‐TNFAIP8 antibody from control and rTNFAIP8‐treated lysates. Red boxes indicate differentially enriched bands. (B) Mass spectrometry results showing STAT1 as the top candidate interacting protein of TNFAIP8 based on peptide coverage and spectral counts. (C) Representative MS/MS spectra confirming STAT1 peptides in the TNFAIP8 pulldown samples. (D) Co‐IP assays in HEK293T cells confirming exogenous interaction between Myc‐TNFAIP8 and Flag‐STAT1. (E) Endogenous Co‐IP showing TNFAIP8–STAT1 binding in CAF cell lysates. (F) GST pull‐down assays were conducted to investigate the direct binding between TNFAIP8 and STAT1. (G) Schematic representation of full‐length and deletion mutants of TNFAIP8 and STAT1 used for domain‐mapping assays. (H, I) Co‐IP assays identifying the minimal regions in TNFAIP8 and STAT1 responsible for their interaction. (J) Western blot showing changes in STAT1 expression in CAFs with TNFAIP8 knockdown or overexpression. (K) Cycloheximide (CHX) chase assay showing STAT1 stability over time in cells with altered TNFAIP8 expression. (L) Quantification of relative STAT1 protein levels after CHX treatment (0–12 h) in different groups (*n* = 3 biological replicates). (M) Proteasome inhibitor MG132, but not lysosome inhibitor chloroquine (CQ), blocks STAT1 degradation induced by TNFAIP8 knockdown. (N) Co‐IP showing that TNFAIP8 promotes STAT1 ubiquitination in HEK293T cells. (O) Ubiquitination screening of STAT1 by TNFAIP8 with indicated types of ubiquitin (K6O, K11O, K27O, K29O, K33O, K48O, and K63O). (P) Co‐IP validation using K48‐only (K48O) and K48‐deficient (K48R) ubiquitin mutants confirming K48‐dependence of TNFAIP8‐mediated STAT1 ubiquitination. Data in (L) are presented as mean ± SD; Significance thresholds: **p < 0.01, ****p < 0.0001; one‐way ANOVA with Tukey's post hoc test.

To investigate the potential mechanism by which TNFAIP8 acts on CAFs and influences fibrosis in PDAC, we performed co‐immunoprecipitation (Co‐IP) combined with mass spectrometry (MS) analysis and identified STAT1 as a top interactor of TNFAIP8 (unique peptides>5, Coverage>10). Silver staining and Co‐IP/MS confirmed STAT1 peptides specifically enriched in the TNFAIP8 pulldown complex (Figure 5A–C). Exogenous and endogenous Co‐IP, Glutathione S‐Transferase (GST) pull‐down further validated the direct interaction between TNFAIP8 and STAT1 (Figure 5D–F). Further molecular mapping assays revealed that the domain2 (residues 50–82) was the main fragment of TNFAIP8 responsible for binding to the domain5 (residues 671–750) of STAT1 (Figure 5G–I). Functionally, TNFAIP8 knockdown increased, whereas its overexpression decreased STAT1 protein levels (Figure 5J). Cycloheximide (CHX) chase assays demonstrated that TNFAIP8 reduced STAT1 stability over time, indicating a role in STAT1 degradation (Figure 5K,L). MG132, but not chloroquine, rescued STAT1 expression under TNFAIP8 knockdown, suggesting proteasome‐mediated degradation (Figure 5M). Moreover, ubiquitination assays showed that TNFAIP8 promoted STAT1 polyubiquitination (Figure 5N), specifically through K48‐linked chains as determined by linkage‐specific ubiquitin mutants (Figure 5O,P). Together, these results indicate that TNFAIP8 interacts with STAT1 and facilitates its proteasomal degradation through K48‐linked ubiquitination.

### 2.6 TNFAIP8 Recruits TRIM21 to Facilitate STAT1 Ubiquitination and Myofibroblastic CAF–Associated Features

**FIGURE 6:**
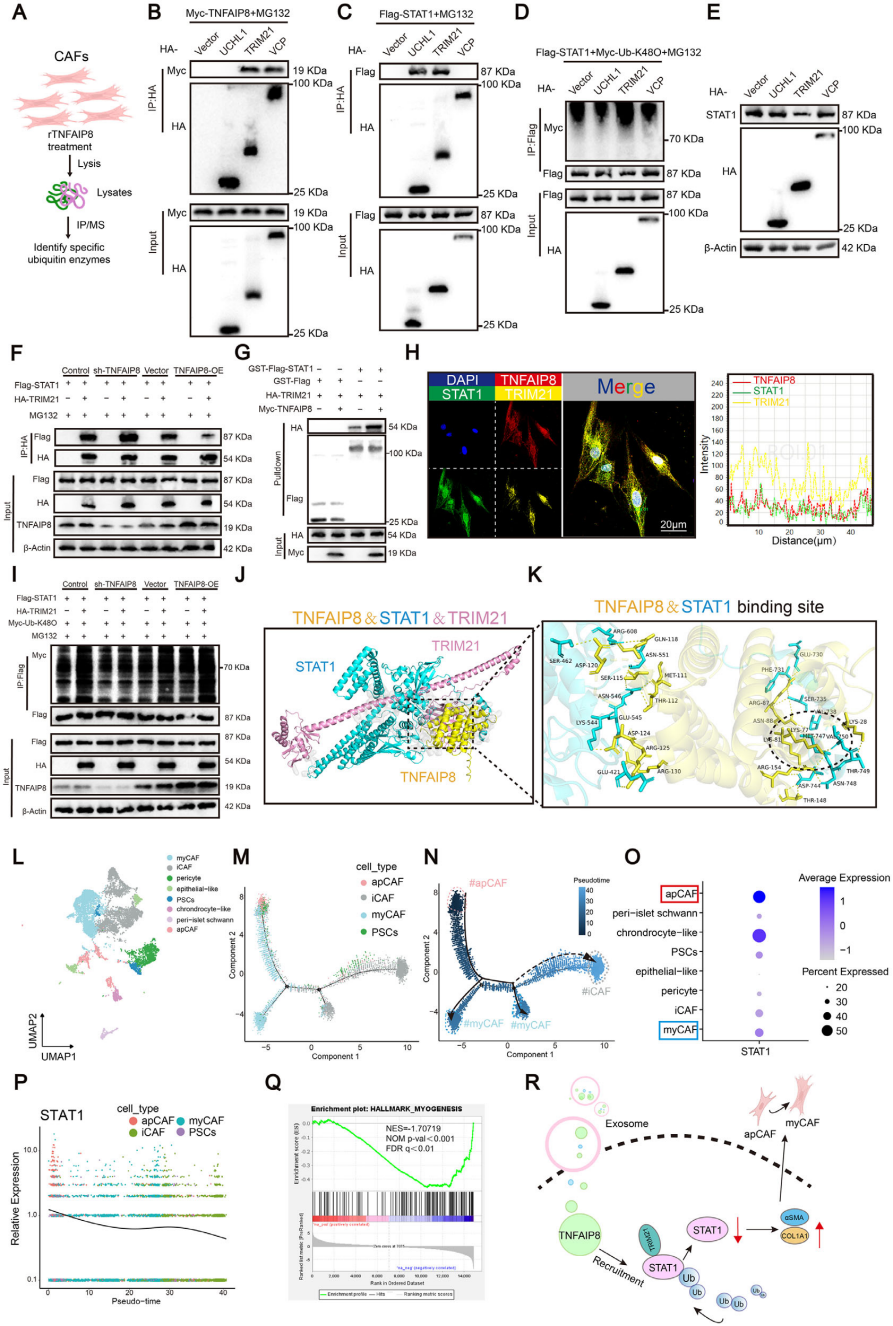
TNFAIP8 recruits TRIM21 to facilitate STAT1 ubiquitination and modulate CAF subtype–associated phenotypes. (A) Schematic of IP/MS workflow to identify TNFAIP8‐binding ubiquitin enzymes in CAFs. (B) The interaction of TNFAIP8, UCHL1, TRIM21, VCP in HEK293T cells was analyzed using IP. (C) The interaction of STAT1, UCHL1, TRIM21, VCP in HEK293T cells was analyzed using IP. (D) K48‐linked ubiquitination of STAT1 in HEK293T cells transfected with UCHL1, TRIM21, and VCP. (E) The protein expression levels of STAT1 in HEK293T cells transfected with UCHL1, TRIM21, and VCP. (F) The interaction of STAT1 and TRIM21 in TNFAIP8 knockdown and overexpression cells. (G) GST pull‐down assay confirming direct binding between TNFAIP8 and TRIM21. (H) IF confocal images showing co‐localization of TNFAIP8, STAT1, and TRIM21 in CAFs. Right panel: fluorescence intensity profiles along the indicated line, demonstrating co‐localization. Scale bar, 50 µm. (I) K48‐linked ubiquitination of STAT1 in TNFAIP8 knockdown and overexpression cells. (J) The simulated 3D triad structures of TNFAIP8, STAT1, and TRIM21 are shown as cartoon models in Yellow, blue, and pink, respectively. (K) The binding sites in the domain2 (residues 49–83) of TNFAIP8 are shown as Yellow stick models, and those in the domain5 (residues 670–750) of STAT1 are shown as blue stick models. (L) UMAP visualization of single‐cell RNA‐seq data from GSE205013, showing the identification and annotation of distinct CAF subtypes, including myCAF, iCAF, and apCAF, together with other stromal. (M) Trajectory inference of CAF populations based on pseudotime analysis. (N) Pseudotime mapping colored by CAF subtype identity, illustrating the relative distribution of apCAF and myCAF states along the trajectory. (O) Dot plot showing expression of STAT1 across CAF subtypes. (P) Dynamic changes in STAT1 expression along pseudotime in different CAF populations. (Q) GSEA enrichment plot indicating MYOGENESIS pathway is significantly suppressed in STAT1‐high expression group. (R) Schematic summarizing TNFAIP8‐mediated STAT1 degradation and its association with myCAF‐like phenotypic features.

Since TNFAIP8 lacks intrinsic ubiquitination activity, we hypothesized that a specific ubiquitin‐modifying enzyme mediates TNFAIP8‐regulated STAT1 expression. Through Co‐IP‐MS analysis UCHL1, TRIM21, and VCP were identified as potential TNFAIP8‐binding partners (Figure 6A). Co‐IP assays in HEK293T cells showed that both TNFAIP8 and STAT1 bind TRIM21, while only TRIM21 significantly enhanced STAT1 K48‐linked ubiquitination and reduced STAT1 protein levels (Figure 6B–E). Co‐IP assays demonstrating interactions between TNFAIP8 and TRIM21 (Figure S8A), and between TRIM21 and STAT1 (Figure S8B) in PDAC cells. IgG was used as a negative control. Moreover, TNFAIP8 knockdown diminished the association between TRIM21 and STAT1, while TNFAIP8 overexpression enhanced this interaction, indicating that TNFAIP8 facilitates TRIM21 binding to STAT1 (Figure 6F). GST pull‐down assays confirmed a direct binding between TNFAIP8 and TRIM21 (Figure 6G). IF further demonstrated the co‐localization of TNFAIP8, TRIM21, and STAT1 in CAFs (Figure 6H). TNFAIP8 knockdown suppressed, whereas TNFAIP8 overexpression promoted, STAT1 K48‐linked ubiquitination (Figure 6I). Furthermore, we simulated the 3D structure of the TNFAIP8/STAT1/TRIM21 complex, and the docking results showed that amino acids LYS‐77 and LYS‐81 in the 50–82 region of TNFAIP8 interacted with amino acids MET‐747, ASN‐748, and VAL‐750 in the 671–750 region of STAT1 (Figure 6J,K). Mutation of these binding sites abolished TNFAIP8–STAT1 interactions, STAT1 ubiquitination, and TRIM21‐mediated degradation, confirming the functional importance of this interface (Figure S8C–E).

Single‐cell RNA‐seq analysis of PDAC samples (GSE205013) revealed pronounced heterogeneity among CAF populations, allowing the identification of distinct CAF subtypes, including myCAF, iCAF, and apCAF (Figure 6L; Figure S8F). Pseudotime trajectory analysis positioned these CAF subsets along a continuous differentiation axis, with apCAFs enriched at early pseudotime and myCAFs predominating at later stages (Figure 6M,N). Consistent with this trajectory, STAT1 expression was highest in apCAFs and progressively decreased along pseudotime toward myCAF states (Figure 6O,P; Figure S8G). Gene set enrichment analysis further demonstrated that STAT1‐high CAFs exhibited suppression of myogenesis‐related programs, indicating an inverse association between STAT1 activity and myCAF‐like fibrotic features (Figure 6Q; Figure S8H). Modulation of TNFAIP8 in CAFs selectively altered CAF subtype–associated gene expression programs. qPCR analysis showed that rTNFAIP8 treatment suppressed apCAF markers (CD74, HLA‐DRA) while promoting myCAF markers (ACTA2, COL1A1), with minimal effects on iCAF markers (IL6, IL8); these effects were partially reversed by proteasome inhibition with MG132 (Figure S8J). Immunoblotting further confirmed that rTNFAIP8 reduced STAT1 and p‐STAT1 levels while increasing myCAF‐associated proteins, including αSMA and COL1A1, an effect attenuated by MG132 (Figure S8K,L). Concordantly, IF quantification demonstrated decreased CD74 and STAT1 signals and enhanced αSMA expression in CAFs following rTNFAIP8 treatment, supporting a TNFAIP8‐driven shift toward a myCAF‐like phenotype through proteasome‐dependent STAT1 regulation (Figure S8M,N). A schematic illustrates how TNFAIP8 recruits TRIM21 to promote STAT1 ubiquitination, supporting myCAF‐associated phenotypic remodeling (Figure 6R).

Collectively, these results identify TRIM21 as a TNFAIP8‐interacting E3 ligase that mediates STAT1 degradation and establish TNFAIP8 as a key driver of CAF subtype plasticity, specifically associated with a bias toward myCAF‐like features relative to apCAF states in PDAC.

### 2.7 TNFAIP8 Knockdown Attenuates Insulin‐Associated PDAC Growth and Restores STAT1 Levels

**FIGURE 7:**
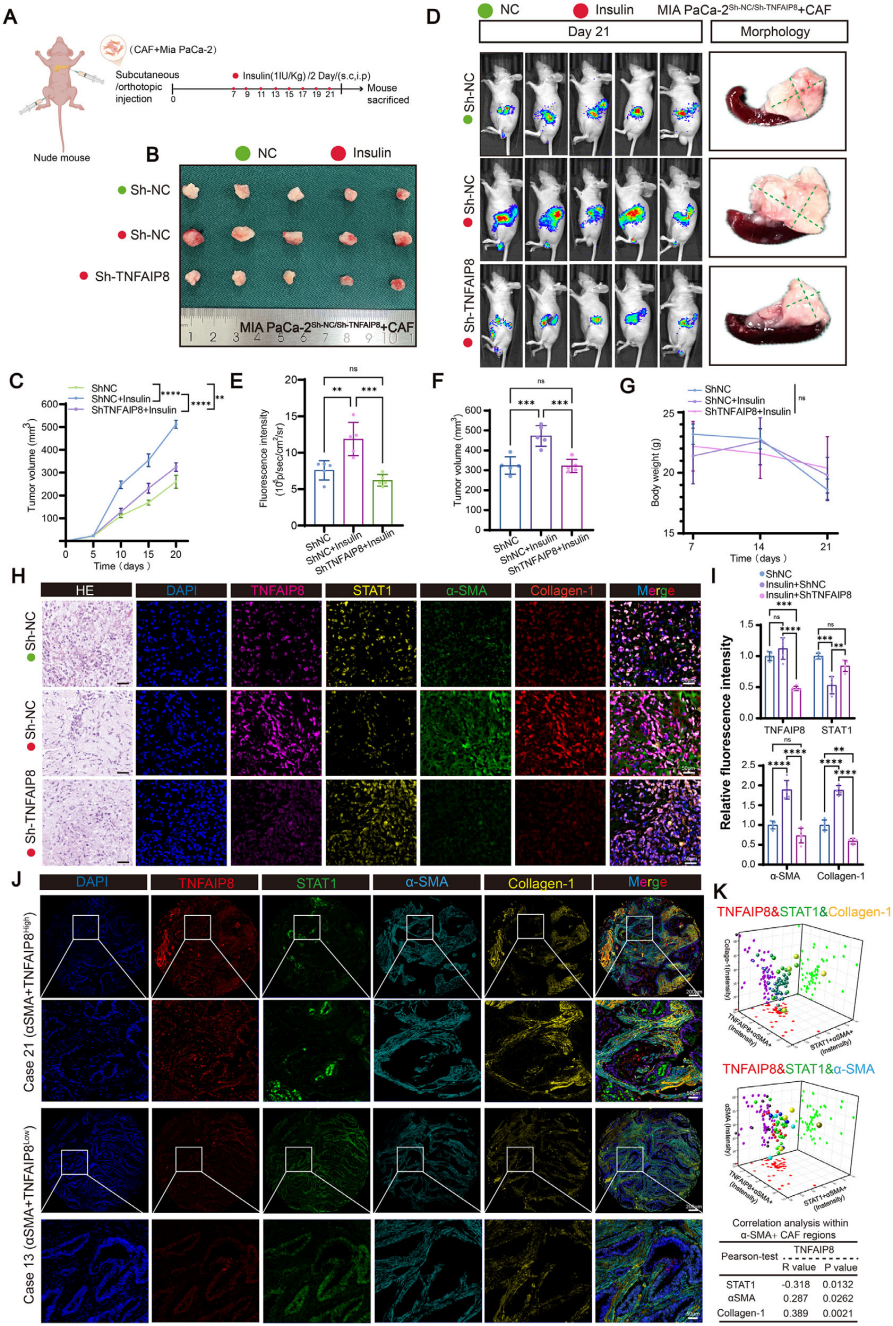
TNFAIP8 knockdown reduces insulin‐driven PDAC tumor growth in vivo. (A) Schematic depicting subcutaneous or orthotopic co‐injection of Mia PaCa‐2 cells and CAFs into nude mice, followed by insulin administration (1 IU/kg, every 2 days, subcutaneous or intraperitoneal injection). (B) Representative images of harvested subcutaneous tumors from mice with Sh‐NC or Sh‐TNFAIP8 Mia PaCa‐2 cells ± insulin treatment. (C) Growth curves of subcutaneous tumor volumes in indicated groups (*n* = 5 mice per group). (D) Representative bioluminescence images (Day 21) and gross morphology of orthotopic tumors in the indicated groups. (E) Quantification of luciferase activity from orthotopic tumors (*n* = 5 mice per group). (F) Tumor volume measurements of orthotopic tumors at endpoint. (G) Body weight of mice recorded during the experimental period. (H) Representative H&E staining and multiplex IF staining of TNFAIP8, STAT1, α‐SMA, and Collagen‐1 in orthotopic tumors from indicated groups. Scale bar, 50 µm. (I) Quantification of relative fluorescence intensities of TNFAIP8, STAT1, α‐SMA, and Collagen‐1 from panel H (*n* = 5 per group). (J) Representative multiplex IF images of human PDAC samples with high (Case 21) or low (Case 13) TNFAIP8 levels in α‐SMA–positive areas, showing TNFAIP8, STAT1, α‐SMA, and Collagen‐1 localization. Scale bar, 50 µm. (K) 3D scatter plots showing correlation analyses among TNFAIP8, STAT1, and stromal markers (Collagen‐1) in PDAC α‐SMA+ tissues (TMA, *n* = 60). Data in (C, E, F, G, I) are presented as mean ± SD; one‐way ANOVA with Tukey's post hoc test. Correlation analyses were conducted using Pearson's correlation coefficient (K). Significance thresholds: *p < 0.05, **p < 0.01, ***p < 0.001.

To investigate the impact of TNFAIP8 on insulin‐driven PDAC progression in vivo, first of all, TNFAIP8 knockdown in MIA PaCa‐2 cells did not significantly affect tumor cell proliferation, clonogenic capacity, or EdU incorporation in vitro (Figure S9A–E). Consistently, orthotopic implantation and in vivo bioluminescence imaging showed no significant differences in tumor growth or signal intensity between control and TNFAIP8‐silenced tumors (Figure S9F–I). These findings indicate that TNFAIP8 does not exert a cell‐autonomous effect on PDAC tumor growth. We performed subcutaneous and orthotopic co‐injection of Mia PaCa‐2 cells and CAFs into nude mice, followed by insulin administration (Figure 7A). TNFAIP8 depletion significantly reduced tumor growth and bioluminescence signals in both models, even under insulin stimulation (Figure 7B–G). Histological analysis of orthotopic tumors showed that TNFAIP8 knockdown attenuated insulin‐induced expression of α‐SMA, and Collagen‐1, revealed STAT1 levels, as demonstrated by H&E and multiplex IF staining (Figure 7H). Quantification further confirmed significantly reduced fluorescence intensities of these markers in TNFAIP8‐silenced tumors (Figure 7I). To validate these findings in human PDAC samples, multiplex IF revealed higher α‐SMA, and Collagen‐1 and lower STAT1 levels in tumors with high TNFAIP8 expression, especially in α‐SMA–positive regions (Figure 7J; Figure S9J). Correlation analysis showed that TNFAIP8 expression correlated positively with stromal fibrosis–associated markers, including Collagen‐1 and α‐SMA, in human PDAC tissues. (Figure 7K).

To sum up, these results indicate that TNFAIP8 promotes insulin‐induced PDAC tumor growth and stromal fibrosis in vivo, potentially by modulating STAT1 and fibrotic marker expression.

### 2.8 LNP‐Encapsulated shTNFAIP8 as a Potential Therapeutic Strategy Associated With Reduced PDAC Progression and Stromal Fibrosis

**FIGURE 8:**
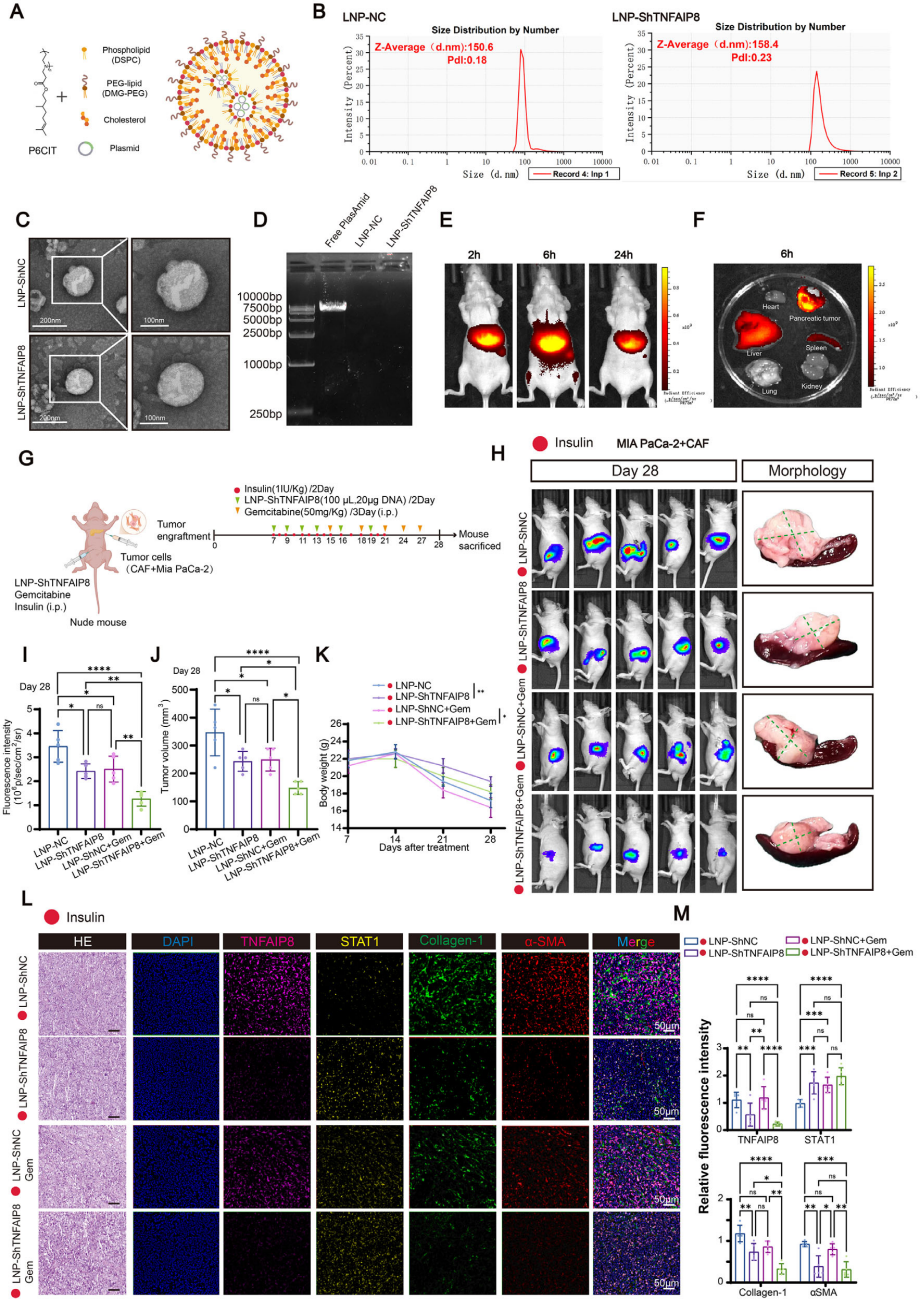
Therapeutic strategy using LNP‐encapsulated shTNFAIP8 plasmid reveals anti‐tumor effects. (A) Schematic illustration of LNP structure composed of phospholipid (DSPC), PEG‐lipid (DMG‐PEG), cholesterol, and encapsulated plasmid DNA. (B) Size distribution and polydispersity index (PDI) of LNP‐NC and LNP‐ShTNFAIP8 determined by dynamic light scattering. (C) TEM images showing spherical morphology of LNP‐NC and LNP‐ShTNFAIP8. Scale bars, 200 nm and 100 nm. (D) Agarose gel electrophoresis analysis of LNP formulations to assess plasmid DNA encapsulation. (E) Fluorescence imaging of DIR‐labeled LNPs in orthotopic tumor‐bearing mice at different time points. (F) Fluorescence images showed the biodistribution of DIR‐labeled LNP in tumors and major organs at 6 h post‐injection. (G) Experimental scheme of in vivo treatments: tumor‐bearing nude mice were administered insulin (1 IU/kg, every 2 days), LNP‐ShTNFAIP8 (100 µL, 20 µg DNA, every 2 days), and/or gemcitabine (50 mg/kg, every 3 days, i.p.). (H) Representative bioluminescence images on Day 28 and gross morphology of orthotopic tumors from each treatment group. (I, J) Quantification of tumor fluorescence intensity and tumor volume in indicated groups on Day 28 (*n* = 5 mice per group). (K) Body weight changes were recorded throughout the experimental period (*n* = 5 mice per group). (L) Representative H&E staining and multiplex IF images of TNFAIP8, STAT1, Collagen‐1, and α‐SMA in orthotopic tumor tissues from the indicated groups. Scale bars, 50 µm. (M) Quantification of IF intensities of TNFAIP8, STAT1, Collagen‐1, and αSMA in tumor tissues from the indicated treatment groups (*n* = 5 per group). Data in (I, J, K, M) are presented as mean ± SD and analyzed by one‐way ANOVA with Tukey's post hoc test. Significance thresholds: ns, not significant; *p < 0.05; **p < 0.01; ****p < 0.0001.

LNP delivery technology represents a revolutionary platform for therapeutic development, enabling efficient delivery of diverse nucleic acid cargos into tumor cells, while improving stability, cellular uptake, and antitumor efficacy. To explore the translational potential of targeting TNFAIP8 in PDAC, we employed LNP technology for nucleic acid delivery. Following the optimized formulation and synthesis method described by Wang et al., which demonstrated efficient pancreas‐targeting via intraperitoneal administration [23], we established a similar LNP‐based delivery system for encapsulating shTNFAIP8 plasmid DNA. The LNPs were prepared using P6CIT, DSPC, DMG‐PEG2000, and Cholesterol (Figure 8A), with particle sizes characterized by dynamic light scattering (DLS) and morphology assessed by TEM (Figure 8B,C). Meanwhile, agarose gel electrophoresis showed that LNP‐NC and LNP‐shTNFAIP8 did not display visible DNA bands compared to the free plasmid, confirming that the shTNFAIP8 plasmid was successfully encapsulated within the LNPs (Figure 8D). DIR‐labeled LNPs exhibited efficient accumulation in orthotopic pancreatic tumors (Figure 8E,F). Gemcitabine dose–response analysis showed that TNFAIP8 knockdown did not significantly alter tumor cell sensitivity to gemcitabine in vitro (Figure S8A). In vivo treatment experiments were performed in orthotopic PDAC models under insulin stimulation to mimic hyperinsulinemic conditions. Mice treated with LNP‐shTNFAIP8 showed significant reductions in tumor bioluminescence intensity and tumor volumes compared to controls, and this effect was further enhanced when combined with gemcitabine (Figure 8G–J). Notably, Body weight declined over time in all groups, but was better maintained in LNP‐ShTNFAIP8–treated mice (Figure 8K). Multiplex IF and histological analyses, together with quantitative fluorescence intensity measurements, confirmed that LNP‐shTNFAIP8 treatment reduced TNFAIP8, α‐SMA, and Collagen‐1 expression in tumor tissues. These effects were further enhanced when combined with gemcitabine, indicating a pronounced attenuation of stromal fibrosis and tumor progression (Figure 8L,M). These results suggest that alleviating tumor fibrosis could potentially enhance the effectiveness of chemotherapeutic drugs against tumor cells. H&E staining of major organs and serum biochemical analyses showed no significant histopathological alterations or hepatotoxicity/nephrotoxicity in LNP‐treated mice, supporting the safety of this therapeutic approach (Figure S8B,C). Furthermore, IHC revealed increased cleaved caspase‐3 and decreased Ki‐67 expression in LNP‐shTNFAIP8+Gem treated tumors, indicating enhanced apoptosis and reduced proliferation (Figure S8D,E).

**FIGURE 9:**
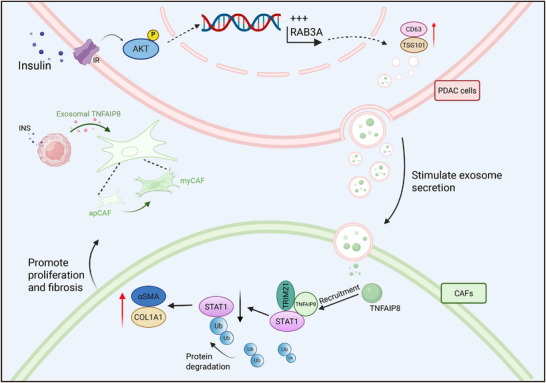
Mechanistic model of insulin‐associated exosomal TNFAIP8 signaling and stromal activation in PDAC.

These data indicate that LNP‐mediated delivery of shTNFAIP8 is associated with reduced PDAC tumor growth and stromal fibrosis and enhanced gemcitabine sensitivity, without evident systemic toxicity, supporting its potential translational relevance. Collectively, this study identifies an insulin‐responsive tumor–stroma regulatory axis in PDAC. Insulin‐mediated activation of the PI3K/AKT–RAB3A pathway enhances exosome secretion and TNFAIP8 transfer to CAFs, where TNFAIP8 promotes STAT1 ubiquitination and proteasomal degradation, contributing to myofibroblastic remodeling, fibrosis progression, and increased chemoresistance (Figure 9).

## 3 Discussion

The growing incidence of pancreatic cancer in the setting of obesity and type 2 diabetes underscores the urgent need to understand how systemic metabolic alterations shape tumor biology [24, 25]. Hyperinsulinemia has been recognized as a risk factor for PDAC, but mechanistic insight into its influence on the tumor microenvironment has been limited [26]. Our study identifies a direct pathway through which insulin signaling promotes desmoplastic progression, linking metabolic dysregulation to stromal remodeling via an insulin‐exosome‐TNFAIP8‐STAT1 axis.

A key advance is the demonstration that insulin activates PI3K/AKT‐RAB3A signaling to drive enhanced secretion of tumor‐derived exosomes enriched in TNFAIP8. This finding adds to the established role of insulin in regulating vesicle trafficking in metabolic tissues. Still, it reveals a tumor‐specific effector‐RAB3A‐that couples insulin signaling to pro‐tumorigenic exosome release. This represents a shift from the prevailing view of hyperinsulinemia primarily as a mitogenic stimulus for cancer cells, positioning it instead as an active modulator of stromal behavior.

We further show that TNFAIP8‐enriched exosomes act in a paracrine fashion on CAFs, where TNFAIP8 recruits the E3 ligase TRIM21 to induce K48‐linked polyubiquitination and degradation of STAT1. STAT1 has been implicated as an anti‐fibrotic factor in other organ systems, but its role in pancreatic cancer‐associated fibrosis has not been established. Our data indicate that STAT1 downregulation is associated with a shift from apCAF toward myCAF‐like phenotypes, accompanied by enhanced extracellular matrix deposition and tumor‐supportive fibrosis. This mechanistic link between metabolic signaling and CAF subtype plasticity adds a new dimension to the understanding of PDAC desmoplasia.

The translational relevance of these findings is underscored by the association between high TNFAIP8 expression, dense stroma, and poor clinical outcomes in patient samples. Importantly, genetic silencing of TNFAIP8 or lipid nanoparticle‐mediated delivery of shTNFAIP8 not only attenuated fibrosis and reduced tumor burden in orthotopic models but also improved gemcitabine efficacy without evident systemic toxicity. These results suggest that targeting TNFAIP8 might represent a viable stromal‐directed strategy, particularly relevant in the large subset of PDAC patients with metabolic comorbidities.

Our study also supports a broader conceptual framework in which systemic metabolic cues are integrated into the tumor microenvironment through exosomal communication. This model may extend to other hyperinsulinemia‐associated malignancies and suggests that metabolic control and stromal targeting could be combined in therapeutic regimens.

Several limitations warrant consideration. While we used patient‐derived samples to establish clinical correlations, prospective validation in larger cohorts is needed to confirm the prognostic value of TNFAIP8. In addition, although we focused on fibroblast reprogramming, TNFAIP8‐containing exosomes may also affect immune or endothelial compartments, which merits investigation. While our integrated mechanistic, transcriptomic, and phenotypic analyses support a TNFAIP8–STAT1–associated shift toward myofibroblastic CAF features, the proposed apCAF‐to‐myCAF transition is inferred rather than directly demonstrated. Definitive confirmation will require in vivo lineage tracing or CAF subtype–resolved single‐cell and spatial approaches. Future studies using such high‐resolution models will be essential to fully delineate CAF state dynamics under hyperinsulinemic conditions. Finally, the durability and pharmacokinetics of LNP‐based TNFAIP8 silencing require further optimization for clinical translation.

Previous studies on hyperinsulinemia in PDAC have largely focused on its direct impact on tumor cell proliferation and metabolism [12, 13, 15, 16], Canonical insulin–INSR signaling has been well established in pancreatic cancer, primarily acting through PI3K–AKT and MAPK pathways [27, 28, 15, 29], with limited attention to its role in shaping the tumor microenvironment [11]. Our findings reveal a distinct mechanism: Transcriptomic analysis revealed that insulin stimulation activates the canonical PI3K/AKT/FoxO signaling axis in tumor cells and concurrently upregulates RAB3A, a key regulator of vesicle. RAB3A expression was significantly correlated with exosome markers such as CD63 and TSG101 in PDAC samples, and functional assays confirmed its role in promoting exosome release and CAF activation. In both in vitro and in vivo models, RAB3A overexpression amplified insulin‐induced tumor growth and stromal fibrosis, whereas pharmacologic blockade of exosome secretion abrogated these effects. Notably, while insulin is known to regulate vesicle trafficking in metabolic tissues such as GLUT4 transport via RAB8A and RAB10 [30, 31]. Our findings identify RAB3A as a tumor‐specific effector linking insulin signaling to exosome release and fibroblast reprogramming in PDAC, consistent with its vesicle‐regulatory role observed in other contexts such as spinal cord injury.

Beyond its tumor cell–intrinsic effects, our study reveals a novel role for exosomal TNFAIP8, induced by insulin stimulation, as a paracrine mediator of fibroblast reprogramming in the pancreatic tumor microenvironment. When packaged into tumor‐derived exosomes, TNFAIP8 was internalized by CAFs and directly bound to STAT1, a key transcriptional regulator of fibroblast phenotype maintenance. Mechanistically, TNFAIP8 acts as an adaptor to recruit the E3 ligase TRIM21, promoting STAT1 degradation through K48‐linked polyubiquitination. Single‐cell RNA‐seq analysis showed that STAT1 is predominantly expressed in apCAFs [32], a subtype associated with immune modulation [33]. Downregulation of STAT1 by exosomal TNFAIP8 is associated with a phenotypic shift toward myCAF‐like features, which are characterized by enhanced fibrogenic programs and desmoplastic remodeling.

Notably, STAT1 has been widely recognized as an anti‐fibrotic factor in multiple organs. In liver fibrosis, its activation promotes stellate cell apoptosis and reduces collagen deposition [34], while in the lung and bladder, STAT1 loss enhances STAT3 signaling and fibrotic progression [35, 36]. These findings suggest that STAT1 restrains fibroblast activation, and its downregulation may contribute to stromal fibrosis. However, the role of STAT1 in pancreatic cancer–associated fibrosis has not been previously elucidated. Our findings identify exosomal TNFAIP8 as a key paracrine mediator associated with CAF phenotypic plasticity and stromal, potentially through modulation of STAT1 ubiquitination and degradation. Importantly, this work is the first to define a complete “insulin–exosome–TNFAIP8–CAF” fibrotic axis, linking metabolic dysfunction to fibroinflammatory progression in PDAC through a clearly defined molecular pathway.

CAFs are increasingly recognized as key regulators of therapeutic resistance in PDAC. Distinct CAF subtypes—myCAFs, iCAFs, and apCAFs—differentially contribute to ECM remodeling, immune suppression, and drug response [37, 38, 39]. For example, CAF‐derived IL‐6 activates the mTOR/4E‐BP1 axis to promote gemcitabine resistance, which can be reversed by the somatostatin analog SOM230 targeting CAF‐specific receptors [40, 41]. CAFs also impair immunotherapy efficacy by restricting T cell infiltration and modulating antigen presentation. Clinically, high CAF infiltration and CAF‐related gene signatures correlate with poor chemotherapy response and reduced survival [42, 43].

Our study suggests that exosomal TNFAIP8 functions upstream of CAF phenotypic remodeling and is associated with a transition toward myofibroblastic CAF features. Fibrotic CAFs drive ECM stiffening, restrict drug delivery, and foster an immunosuppressive niche. By defining the insulin–exosome–TNFAIP8 axis that governs CAF plasticity, we highlight a potential therapeutic target to remodel the stroma and enhance treatment efficacy in PDAC. LNP–mediated delivery platforms have recently shown promise in pancreatic disease by enabling efficient, localized, and non‐invasive nucleic acid delivery [44, 45]. Notably, pancreas‐targeted LNPs carrying IL‐12 mRNA or STING agonists have been shown to activate local immune responses, reprogram the fibrotic microenvironment, and enhance anti–PD‐1 efficacy in PDAC models [23, 46, 47, 48]. In our study, LNP‐based delivery of shTNFAIP8 significantly reduced tumor fibrosis and enhanced gemcitabine sensitivity, supporting the feasibility of targeting the insulin–exosome–TNFAIP8 axis in vivo. Together, th ese findings suggest that stromal‐targeted nanotherapies may complement existing chemotherapy or immunotherapy approaches and could help address drug resistance in PDAC.

Importantly, our findings should be interpreted within the broader context of CAF heterogeneity and functional plasticity in PDAC. Increasing evidence indicates that αSMA‐high myCAFs are not uniformly tumor‐promoting; in certain settings, they can restrain tumor progression by maintaining stromal structure and limiting epithelial plasticity. Indeed, depletion of myCAFs has been shown to paradoxically accelerate tumor growth and worsen survival in preclinical models, highlighting the context‐dependent nature of CAF function [6, 49]. Current single‐cell and spatial studies support a model in which CAFs exist along a dynamic continuum, with myCAF and iCAF states being interconvertible in response to microenvironmental cues such as TGF‐β signaling, inflammatory factors, and mechanical stress [50, 51]. Thus, CAF behavior is determined less by static marker expression and more by state‐specific activation programs shaped by tumor‐derived signals.

Within this framework, our data do not support indiscriminate stromal targeting or a uniformly pro‐tumorigenic role of myCAFs. Rather, they indicate that under hyperinsulinemic metabolic conditions, insulin/INSR signaling selectively reprograms the tumor–stroma axis. Specifically, insulin enhances tumor cell secretion of TNFAIP8, promoting myCAF transition, extracellular matrix remodeling, and fibrotic stromal reinforcement, which in turn contributes to increased chemoresistance. These findings support a more nuanced therapeutic strategy aimed at selectively modulating metabolically driven, pro‐fibrotic CAF programs rather than globally ablating the stromal compartment.

In summary, our study delineates an insulin–exosome–TNFAIP8‐STAT1 signaling axis that provides a mechanistic framework linking metabolic dysregulation to fibroinflammatory remodeling in PDAC. By elucidating how hyperinsulinemia is associated with CAF subtype reprogramming, enhanced stromal fibrosis, and reduced therapeutic responsiveness, our findings suggest that TNFAIP8 may represent a potential target for stroma‐directed therapeutic strategies. These results suggest that integrating metabolic modulation with selective stromal targeting may represent a complementary strategy to improve therapeutic outcomes in PDAC, a malignancy long resistant to conventional therapies.

## 4 Methods

### 4.1 Human Specimens

A total of 60 tumor samples and paired adjacent normal tissues were collected from patients diagnosed with PDAC between 2018 and 2019 at the First Affiliated Hospital of Nanjing Medical University. Fresh tissues were immediately frozen at −80°C after surgery for long‐term preservation. This study was ratified by the Human Ethics Committee of the First Affiliated Hospital of Nanjing Medical University (2021‐SRFA‐402). Each patient was informed about the study and signed a consent form. Patients who have received neoadjuvant chemotherapy, radiotherapy, or immunotherapy were excluded.

### 4.2 Cell Lines and Culture

Human PDAC cell lines AsPC‐1 (RRID: CVCL_0152), PANC‐1 (RRID: CVCL_0480), BxPC‐3 (RRID: CVCL_0186), CFPAC‐1 (RRID: CVCL_1119), and MIA PaCa‐2 (RRID: CVCL_0428), as well as HEK293T cells (RRID: CVCL_0063), were obtained from the Cell Bank of the Chinese Academy of Sciences (Shanghai, China). Primary CAFs were isolated from surgically resected PDAC tissues from four patients with informed consent and approval from the institutional ethics committee. All cell lines were authenticated by short tandem repeat (STR) profiling and tested negative for mycoplasma contamination before use. PDAC cell lines were maintained in DMEM (Wisent) supplemented with 10% fetal bovine serum (FBS, Wisent) and 1% penicillin–streptomycin in a humidified incubator at 37°C with 5% CO_2_. Primary CAFs were cultured in DMEM/F12 (Wisent) containing 10% FBS and 1% penicillin–streptomycin under the same conditions.

### 4.3 Serum Insulin and C‐Peptide Measurement

Fasting serum insulin and C‐peptide levels were measured using electrochemiluminescence immunoassays (ECLIA) on the Roche Elecsys platform. Insulin concentrations were determined with the Elecsys Insulin assay, and C‐peptide levels were quantified using the Elecsys C‐Peptide assay, according to the manufacturer's instructions. All samples were analyzed in duplicate, and quality controls provided by the manufacturer were included in each run.

### 4.4 Origin, Isolation, and Establishment of CAF Lines

The isolation, establishment, and characterization of the CAF cell lines were performed in accordance with ref [52, 53]. Briefly, Fresh tissue was obtained from residual pancreatic adenocarcinoma specimens from patients undergoing primary surgical resection at the Pancreas Center, the First Affiliated Hospital of Nanjing Medical University. Freshly resected tumor tissues were transferred to the laboratory under sterile conditions and rinsed three times with cold 0.9% NaCl or PBS to remove blood and fat. The tissues were cut into small blocks (1 × 1 × 1 mm^3^) and washed until the supernatant was clear. Tissue blocks (5–10 per well) were placed in 6‐well plates and allowed to adhere by partial drying. Subsequently, 2 mL of DMEM/F12 medium (Wisent) supplemented with 10% FBS, and 1% penicillin–streptomycin was gently added. Plates were incubated in a humidified 5% CO_2_ incubator at 37°C without disturbance to allow fibroblasts to migrate from the tissue edges. Medium was refreshed every 3 days. Outgrowing fibroblast‐like cells were typically observed within 5–10 days, reaching 90% confluence in 2–3 weeks, after which they were trypsinized and expanded, cells were trypsinized and passaged 1:2. Cell purity was determined by IF for αSMA FAP, and Vimentin, and the morphology (spindle‐shaped cells with cytoplasmic extensions) was assessed.

### 4.5 Single‐Cell RNA Sequencing (scRNA‐Seq) Data Collection and Analysis

Publicly available scRNA‐seq datasets of human PDAC were retrieved from the Gene Expression Omnibus (GEO) under accession numbers GSA:CRA001160 and GSE205013 [54, 55, 56]. For bulk RNA‐seq data from TCGA‐PAAD, stromal scores were calculated using the ESTIMATE algorithm to infer overall stromal enrichment based on predefined stromal and ECM‐related gene signatures [57]. TCGA‐PAAD samples were rigorously curated by manual review of clinical and pathological annotations, and cases with non‐conventional PDAC histologies (including neuroendocrine, adenosquamous, undifferentiated, colloid, and other variant subtypes) were excluded. After filtering, 147 histologically confirmed PDAC samples were retained for downstream analyses and compared with normal pancreatic tissues from the GTEx cohort to ensure tumor‐type specificity and minimize histological heterogeneity. For single‐cell RNA‐seq data, stromal activity was quantified using a CAF/ECM gene module scoring approach implemented with Seurat's AddModuleScore, incorporating canonical CAF and fibrosis‐related gene sets [58]. The sequencing data were processed using the Seurat (v4.4.0) pipeline in R. Cells with fewer than 200 or more than 7000 detected genes, and those with mitochondrial gene content exceeding 10% were excluded, and doublets were removed via DoubletFinder. Batch effects were corrected using BBKNN [59]. Data were normalized, log‐transformed, and scaled for downstream analyses. Principal component analysis was applied for dimensionality reduction, followed by Uniform Manifold Approximation and Projection (UMAP) for visualization of cellular heterogeneity.

Major PDAC cell populations, including ductal cells, fibroblasts, endothelial cells, myeloid cells, T/NK cells, B cells, plasma cells, and mast cells, were identified based on canonical marker genes. Fibroblast clusters were further reclustered to define cancer‐associated fibroblast (CAF) subtypes, including myCAF, iCAF, and apCAF, as well as stromal subtypes such as pericytes, pancreatic stellate cells, chondrocyte‐like cells, and peri‐islet Schwann cells. The distribution of STAT1 expression across CAF subtypes was assessed using UMAP and dot plot visualization.

Cell–cell communication analysis was conducted with CellChat (v1.5) to infer ligand–receptor interaction networks between epithelial tumor cells (ETCs) and stromal or immune populations. Gene set enrichment analysis (GSEA, v4.3.2) was performed to compare pathway activities between STAT1^High^ and STAT1^Low^ CAFs, demonstrating that the MYOGENESIS pathway was significantly suppressed in STAT1‐high fibroblasts.

All scRNA‐seq analyses were visualized using Seurat (v5), ggplot2, and Complex Heatmap, and the processed data were further applied for ligand–receptor network construction and functional enrichment studies.

### 4.6 Isolation and Identification of Exosomes

Following the collection of the conditioned medium, the CM was collected and centrifuged in the subsequent order [60]: 300 × g, 15 min; 2000 × g, 15 min; 10 000 × g, 30 min; and 1 00 000 × g, 90 min at 4°C for ultracentrifugation (Beckman Coulter, Germany). Exosomes were retrieved from the sediment and were reconstituted in phosphate‐buffered saline. TEM was used for the visualization of exosomes. Through nanoparticle tracking analysis, particle concentration and size were examined (NTA; ZetaView, PMX, Germany). Exosome markers (CD63, TSG101) were detected by Western blotting. Cellular uptake of exosomes was validated using IF with PKH26 dye (Umibio, Shanghai, China), and observed under a laser scanning confocal microscope LSM780 (Zeiss, Oberkochen, Germany).

### 4.7 Quantitative Real‐Time PCR (qRT‐PCR)

Total RNA was extracted from cells and tissues using Trizol reagent (Vazyme, Nanjing, China) and reverse‐transcribed into cDNA with the HiScript III All‐in‐one RT SuperMix (Vazyme, China). qRT‐PCR was performed using ChamQ SYBR qPCR Master Mix (High ROX Premixed) (Vazyme, China). Relative gene expression was calculated by the 2^–ΔΔCt^ method with β‐Actin as the internal control. Primer sequences are listed in Table S1.

### 4.8 Western Blot

Proteins were separated using 10% or 12.5% SDS–PAGE gels prepared with the PAGE Gel Fast Preparation Kit (Epizyme, Shanghai, China) and then transferred onto PVDF membranes (Roche, Shanghai, China). Membranes were blocked with protein‐free rapid blocking buffer (Epizyme) for 40 min at room temperature, followed by incubation with primary antibodies overnight at 4°C. After washing, membranes were incubated with the secondary antibodies for 2 h. Protein signals were detected using enhanced chemiluminescence reagents (Epizyme) and visualized with a bio‐imaging system. The list of primary antibodies is provided in Table S2.

### 4.9 Immunohistochemistry (IHC)

IHC was performed as previously described [52]. Briefly, tissue sections were deparaffinized, rehydrated, and subjected to antigen retrieval, followed by incubation with primary antibodies against Insulin, CCT6A, RAB3A, COL1A1, α‐SMA, and Ki67. The staining intensity was evaluated. Details of the primary antibodies are provided in Table S2.

### 4.10 Immunofluorescence (IF)

Tumor tissues and cultured cells were prepared following standard protocols and blocked with 10% BSA. Samples were incubated with the indicated primary antibodies overnight at 4°C, followed by fluorophore‐conjugated secondary antibodies for 2 h at room temperature. Nuclei were counterstained with DAPI. Images were acquired using a Thunder Imager super‐resolution confocal microscope (Leica Microsystems) and analyzed with ImageJ software. Details of the antibodies are provided in Table S2.

### 4.11 Cell Proliferation Assays

The proliferation of PANC‐1 and MiaPaca‐2 cells was studied using the cell counting kit‐8 (CCK‐8, Dojindo, Japan) and 5‐ethynyl‐2′‐deoxyuridine assay (EdU, Beyotime, China) assays, as described in our previous studies [61].

### 4.12 Cell Apoptosis Detection Assay

Cell apoptosis was evaluated using the Annexin V‐APC/7‐AAD Apoptosis Detection Kit (Multi Sciences, China) following the manufacturer's instructions. Briefly, cells were harvested according to the experimental design and washed twice with pre‐cooled PBS. A total of 2 × 10^5^ cells, including those in suspension, were resuspended in 500 µL of 1× Binding Buffer. Subsequently, 5 µL of Annexin V‐APC and 10 µL of 7‐AAD were added to each sample, followed by gentle mixing and incubation at room temperature in the dark for 5 min. Apoptotic cells were immediately analyzed using a flow cytometer (Beckman Coulter, USA).

### 4.13 ELISA

CM and Exosome from the cultured PANC‐1 and MIA PaCa‐2 were collected. The cell supernatant was centrifuged at 1000 g for 10 min to remove particles and polymers. ELISA assays were performed using relevant ELISA kits [TNFAIP8 (Jingmei, China)] according to the manufacturer's instructions.

### 4.14 Cell Transfection

Lentiviral vectors encoding target genes, shRNAs, and corresponding empty vectors were designed and produced by TransheepBio (Shanghai). Transfections were performed using Lipofectamine 3000 (Invitrogen, CA, USA) according to the manufacturer's instructions. Stable cell lines were generated by selection with 5 µg/mL puromycin (MedChemExpress, MCE) for 2 days. The shRNA sequences used are listed in Table S2.

### 4.15 Co‐Immunoprecipitation (Co‐IP) Assay

Cells were transfected with the indicated plasmids and lysed in ice‐cold IP lysis buffer (Beyotime, Shanghai, China). After centrifugation to remove debris, supernatants containing target proteins were incubated with the appropriate primary antibody or anti‐tag antibody overnight at 4°C to form immune complexes. The complexes were then captured using protein A/G magnetic beads (Santa Cruz Biotechnology) for 2 h at 4°C with gentle rotation. Beads were washed twice with IP lysis buffer and once with pure water, followed by elution in 1× SDS loading buffer and boiling for 10 min. The immunoprecipitated proteins were analyzed by western blot or subjected to MS [61]. Details of primary antibodies are provided in Table S2.

### 4.16 Immunoprecipitation Coupled With Mass Spectrometry (IP‐MS)

Primary CAFs were treated with recombinant TNFAIP8 protein and lysed in ice‐cold IP buffer (Beyotime, Shanghai, China). Cell lysates were incubated overnight at 4°C with the indicated primary antibody and protein A/G agarose beads (Santa Cruz Biotechnology) to form immunocomplexes. After extensive washing, the bound proteins were eluted, resolved by SDS‐PAGE, and visualized using silver staining [62]. Distinct protein bands were excised, digested, and analyzed by liquid chromatography–mass spectrometry (LC‐MS) at Nanjing Jiangbei New District Biomedicine Public Service Platform.

### 4.17 Glutathione S‐Transferase (GST) Precipitation Assays

HEK293T cells were transfected with plasmids encoding GST‐tagged proteins or the GST control. After 48 h, cells were lysed in ice‐cold lysis buffer supplemented with a complete protease inhibitor cocktail. Cell lysates were incubated with Glutathione Sepharose 4FF beads (Bestchrom, Shanghai, China) at 4°C for 3 h with gentle rotation. Beads were washed three times with lysis buffer to remove nonspecific proteins and then eluted by boiling in SDS loading buffer at 95°C for 10 min. The pulled‐down proteins were analyzed by western blotting according to standard procedures.

### 4.18 Ubiquitination Assay

Cells were transfected with the indicated plasmids and subsequently treated with 10 µm MG132 (Sigma) for 6 h to inhibit proteasomal degradation. Cell lysates were prepared using IP lysis buffer (Beyotime, Shanghai, China), followed by sonication and centrifugation according to the manufacturer's protocol. The supernatants were collected, and ubiquitination levels were assessed by WB using the indicated antibodies [62].

### 4.19 Molecular Docking

To investigate the protein‐protein interactions between the human TNFAIP8 (UniProt: O95379), STAT1 (UniProt: P42224), and TRIM21 (UniProt: P19474) proteins, docking was simulated using CoDockPP software, and the model with the highest confidence level was selected from candidate complexes based on the docking score. All structural models were displayed using the PyMol software (https://pymol.org/2/).

### 4.20 Animal Models

All animal experiments were approved by the Institutional Animal Care and Use Committee of Nanjing Medical University (IACUC‐2412009). Six‐week‐old male BALB/c nude mice were purchased from GemPharmatech (Nanjing, China) and maintained under specific pathogen‐free conditions with ad libitum access to food and water.

For subcutaneous and orthotopic pancreatic tumor models, luciferase‐labeled transfected PDAC cells (PANC‐1, MIA PaCa2) were mixed with primary CAFs at a 1:3 ratio and injected either subcutaneously into the flank or directly into the pancreas of anesthetized mice. Insulin (1 IU/kg; Sigma–Aldrich, Cat# I9278) was administered every 2 days via subcutaneous or intraperitoneal injection starting 7 days post‐inoculation [63, 64, 65]. Tumor growth was monitored by caliper measurements and bioluminescence imaging (all the mice received an intraperitoneal injection of D‐luciferin (≥98%, Macklin, Cat# D812647), and were imaged 10 min later with an IVIS 100 Imaging System (Xenogen, Hopkinton, MA, USA) for biological imaging), Subcutaneous tumor volume was measured 5 Days. All the mice were sacrificed three/four weeks post‐injection, after which tumors were excised and subjected to H&E, IHC, IF staining.

For therapeutic studies, orthotopic tumor–bearing mice received LNP‐shTNFAIP8 (100 µL containing 20 µg DNA) via intraperitoneal injection every 2 days, and/or gemcitabine (50 mg/kg, i.p.) every 3 days [47]. After 28 days, the mice were sacrificed, the mice tumor, major organs, and blood samples were immediately collected for further analysis.

### 4.21 Multiplex IF Quantification and Spatial Analysis

Multiplex IF images were quantitatively analyzed using the HALO image analysis platform (Indica Labs, USA) [66]. Positive signal quantification, area‐based measurements, and spatial enrichment analyses were performed as follows.

Tumor and stromal regions were manually delineated on whole‐slide images according to histopathological criteria. All subsequent quantifications were conducted using the positive area analysis module within the HALO platform. Fluorescence channels corresponding to individual markers and signal thresholds were iteratively optimized to ensure accurate identification of positive signals. Identical threshold settings were applied across all sections for the same marker within a given experiment to ensure consistency. Nuclei were identified based on DAPI staining, and cytoplasmic regions were computationally expanded from nuclear masks. The software then calculated multiple parameters, including positive signal intensity (mean fluorescence intensity), positive area. For markers with broad or regionally distributed expression, positive area/total tissue area and mean fluorescence intensity were used as primary readouts to reflect signal enrichment.

For spatial association analyses, α‐SMA positive regions were selected as surrogates of stromal regions ROIs for downstream analyses [67], within which the fluorescence intensities of CK19, INSR, RAB3A, TNFAIP8, STAT1, CD74, and Collagen‐1 were quantified. Co‐localization‐related measurements were therefore interpreted as regional enrichment within marker‐defined ROIs.

All measurements were performed on multiple high‐power fields per sample, and quantitative results were exported for downstream statistical analysis.

### 4.22 Preparation and Characterization of LNPs

The LNPs were synthesized as previously described. ## Briefly, P6CIT (self‐synthesized), cholesterol (MCE, USA), DSPC (MCE, USA), and DMG‐PEG2000 (Avanti Polar Lipids, USA) were dissolved in absolute ethanol at a weight ratio of 18:8:8:3. ShTNFAIP8 plasmid DNA was dissolved in 25 mm sodium acetate buffer (pH 4.0) to a final concentration of 0.1 mg/mL. The ethanolic lipid phase and aqueous plasmid phase were rapidly mixed at a 1:3 (v/v) ratio to achieve a lipid‐to‐DNA weight ratio of 10:1, allowing spontaneous self‐assembly of LNPs. The resulting suspension was incubated at room temperature for 10 min and then dialyzed overnight against PBS (pH 7.4) using a 50 kDa MWCO dialysis membrane to remove residual ethanol and unencapsulated plasmids [23].

The obtained LNPs were sequentially extruded through 200 nm polycarbonate membranes (Whatman, USA) to ensure uniform particle size and subsequently concentrated using 100 kDa ultrafiltration centrifugal tubes (Millipore, USA). The hydrodynamic diameter, polydispersity index (PDI), and zeta potential of LNPs were characterized by DLS using a Zetasizer Nano ZS (Malvern, UK). Transmission electron microscopy (TEM, JEM‐1200EX, JEOL, Japan) was performed to visualize the morphology of the LNPs.

### 4.23 Biodistribution of LNPs

In order to investigate the biodistribution of LNPs, DiR dye (MCE, USA) was incorporated into the lipid mixture during the initial step of LNP preparation to fluorescently label the nanoparticles. Orthotopic pancreatic tumor–bearing BALB/c nude mice were intraperitoneally (I.P.) injected with DiR‐labeled LNPs at a plasmid dose of 20 ug/100 uL. Whole‐body fluorescence images were acquired at the indicated time points using an in vivo imaging system (IVIS, PerkinElmer, USA). After imaging, major organs and pancreatic tumor tissues were excised for ex vivo fluorescence imaging to evaluate the organ‐level distribution of LNPs.

### 4.24 Statistical Analysis

All statistical analyses were performed using SPSS v26.0 (SPSS, Inc., NY, USA), GraphPad Prism 8.0 (GraphPad Software, CA, USA), and OriginPro 9.1 (OriginLab, Northampton, MA, USA). Overall survival (OS) and disease‐free survival (DFS) curves were generated using the Kaplan–Meier method, and differences between groups were evaluated by the log‐rank (Mantel–Cox) test. Sample sizes were determined based on the signal level and consistency observed across groups. All experiments were conducted at least in triplicate, and results are presented as mean ± standard deviation (SD). Pearson's correlation analysis was applied to assess the relationships between variables. Comparisons between two groups were performed using a two‐tailed Student's *t*‐test, while one‐way or two‐way ANOVA followed by Tukey's post hoc test was used for multiple group comparisons. A *p*‐value< 0.05 was considered statistically significant (n.s. = not significant, *p < 0.05, **p < 0.01, ***p < 0.001 and ****p < 0.0001).

## Author Contributions

Z.L., L.C., T.W., and H.J. contributed to data acquisition, data analysis, statistical analysis, and manuscript writing. H.W., M.L., G.X., C.X., H.Y., C.L., C.L., H.Z., and F.S. contributed to data acquisition and reviewed the manuscript. L.Y., J.Y., and Y.M. acquired funding, carried out data acquisition, data analysis, and reviewed the manuscript. L.Y. supervised the study. All authors read and approved the final version of the manuscript. Y.M. is responsible for the overall content as guarantor.

## Conflicts of Interest

The authors declare no conflicts of interest.

## Supporting information




**Supporting File 1**: advs74468‐sup‐0001‐TableS1‐S6.docx.

**Supporting File 2**: advs74468‐sup‐0002‐FigureS1‐S10.docx.

## Data Availability

The data that support the findings of this study are available on request from the corresponding author. The data are not publicly available due to privacy and ethical restrictions.
