# Supplementary material for: An Insulin‐Exosome‐TNFAIP8 Axis Drives Stromal Fibrosis and Therapeutic Resistance in Pancreatic Cancer
Source: Adv Sci (Weinh). 2026 Feb 19;13(24):e15606. doi: 10.1002/advs.202515606 (PMC13116271; doi:10.1002/advs.202515606)
Supplement: Supplementary file 1 — Supporting File 1: advs74468‐sup‐0001‐TableS1‐S6.docx. [file ADVS-13-e15606-s002.docx]

**Supplementary Table**

**Table S1. Sequences of qRT-PCR primers, shRNAs in this study.**

**1.List of primers in the study**

| **Name** | **Sequence** | **Supplier** |
| --- | --- | --- |
| RAB3A-F | CGCTATGCTGACGACTCGTTCA | Tsingke Biotech |
| RAB3A-R | GGTAGTATGCGGTGGTGATGGT | Tsingke Biotech |
| TNFAIP8-F | CGTGGTCAGTTTCCATCAGGTG | Tsingke Biotech |
| TNFAIP8-R | CGTCCATGTGACTTGGCAGTGA | Tsingke Biotech |
| TNFAIP8-F | GCCGTTCAGGCACAAAAGA | Tsingke Biotech |
| TNFAIP8-R | GCACCTCACTACTTGTGTCGTCTATT | Tsingke Biotech |
| STX1A-F | TGGAGAACAGCATCCGTGAGCT | Tsingke Biotech |
| STX1A-R | CCTCTCCACATAGTCTACCGCG | Tsingke Biotech |
| ATP6V1C2-F | GCCGCCTACAACACTCTGAAGA | Tsingke Biotech |
| ATP6V1C2-R | CGATGACCAGAAGTGTGACGAG | Tsingke Biotech |
| SLC6A7-F | CACCTTTGCCTCCTACAACACG | Tsingke Biotech |
| SLC6A7-R | AGCACGGAGAAGATGGCAAAGC | Tsingke Biotech |
| VAMP2-F | CTCCAAACCTCACCAGTAACAGG | Tsingke Biotech |
| VAMP2-R | AGCTCCGACAGCTTCTGGTCTC | Tsingke Biotech |
| GAPDH -F | GGACCTGACCTGCCGTCTAG | Tsingke Biotech |
| GAPDH -R | GTAGCCCAGGATGCCCTTGA | Tsingke Biotech |
| β-Actin-F | CATGTACGTTGCTATCCAGGC | Tsingke Biotech |
| β-Actin-R | CTCCTTAATGTCACGCACGAT | Tsingke Biotech |

**2. List of short hairpin RNAs (shRNAs) sequence**

| sh-TNFAIP8 #1 | Sense | 5’-AGTCACATGGACGGGTTAATA -3’ |
| --- | --- | --- |
|  | Antisense | 5’-TATTAACCCGTCCATGTGACT-3’ |
| sh-TNFAIP8 #2 | Sense | 5’-GTTTCCATCAGGTGGATTATA-3’ |
|  | Antisense | 5’-TATAATCCACCTGATGGAAAC-3’ |
| sh-TNFAIP8 #3 | Sense | 5’-CATCAAGCTGGCCATTCTTTA-3’ |
|  | Antisense | 5’-TAAAGAATGGCCAGCTTGATG-3’ |
| sh-NC | Sense | 5’-GATTCTCCGAACGTGTCACGT-3’ |
|  | Antisense | 5’-ACGTGACACGTTCGGAGAATC-3’ |

**Table S2: List of antibodies in the study.**

| **Name** | **Citation**  PubMed ID | **Supplier** | **Cat no.** | **Clone no.** |
| --- | --- | --- | --- | --- |
| Insulin | 25259810,  36109708,  31485878 | Proteintech | 15848-1-AP | Polyclonal |
| Collagen-1 | 37801199,  37723658,  37817677 | Abcam | ab138492 | EPR7785 |
| CD63 | 36224027,  36171212,  36161709 | Proteintech | 25682-1-AP | Polyclonal |
| TSG101 | 37667547,  38012335,  39039505 | Abcam | ab125011 | EPR7130(B) |
| Calnexin | 32999434,  32999453,  36170811 | Proteintech | 10427-2-AP | Polyclonal |
| β-Actin | 36180975,  36247302,  34867437 | Proteintech | 20536-1-AP | Polyclonal |
| RAB3A | 30237463,  36220098,  36652260 | Proteintech | 15029-1-AP | Polyclonal |
| P-AKT (Ser473) | 34658873,  36139502,  36095961 | Proteintech | 28731-1-AP | Polyclonal |
| AKT | 36178125.  36180975,  36180975 | Proteintech | 10176-2-AP | Polyclonal |
| TNFAIP8 | 34625422,  34901263,  33376549 | Abcam | ab195810 | EPR10058(3) |
| TNFAIP8 | 36288730,  33227392,  33707587 | Proteintech | 15790-1-AP | Polyclonal |
| STAT1 | 36371909,  36643029,  36915717 | Abcam | ab109320 | EPR4407 |
| TRIM21 | 36749630,  37249651,  37864041 | Abcam | ab207728 | EPR20290 |
| Ub | 31575039,  26309161,  34562065 | Proteintech | 10201-2-AP | Polyclonal |
| CD74 | 34391782 | Abcam | ab108393 | EPR4064 |
| INSR | 37497063,  37608294,  33437372 | Abcam | ab227831 | EPR22167 |
| CK19 | 36843847,  36933093,  37096042 | Abcam | ab76539 | EPR1579Y |
| Ki67 | 38063204,  38092758,  38096029 | Abcam | ab92742 | EPR3610 |
| Cleaved Caspase-3 | 40335932,  40331481,  40328251 | Proteintech | 25128-1-AP | Polyclonal |
| Anti-β-actin | 35104170,  35210391,  35467477 | Abcam | ab8226 | mAbcam 8226 |
| Anti-Flag | 26928300,  26871431,  27601467 | MBL | M185-3L | FLA-1 |
| Anti-HA | 26405199,  28055019,  23623749 | MBL | M180-3 | TANA2 |
| Anti-Myc | 20159986,  19763945,  21610094 | MBL | M047-3 | PL14 |
| Anti-His | 21413013,  30598506,  27829222 | MBL | D291-3 | OGHis |
| Goat anti-rabbit IgG | 37408039,  37428798,  37414755 | Jackson | #111-035-003 | Polyclonal |
| α-SMA | 30224759,  36343209,  31915373 | Proteintech | 14395-1-AP | Polyclonal |

**Table S3: List of Organisms in the study.**

| **Name** | **Citation** | **Supplier** | **Sex** | **Age** |
| --- | --- | --- | --- | --- |
| BALB/c nude mice | 37015754,  36907560 | GemPharmatech (Nanjing, China) | male | 6-8 weeks |

**Table S4: List of Biological samples in the study.**

| **Description** | **Source** | **Identifier** |
| --- | --- | --- |
| Human PDAC tissues | The First Affiliated Hospital of Nanjing Medical University | The Research Ethics Committee of the First Affiliated Hospital of Nanjing Medical University |

**Table S5: List of Software in the study.**

| **Software name** | **Manufacturer** | **Version** |
| --- | --- | --- |
| Image J | National Institutes of Health | 1.8.0 |
| GraphPad Prism | GraphPad Software | 9.5 |
| FlowJo | Tree Star Inc. | 10.8.1 |
| SPSS | IBM SPSS | 26.0 |
| Image Lab | Bio-Rad | 3.0 |
| R | R Foundation | 4.2.1 |
| RStudio | Posit | 2022.07.2 |
| OriginPro | OriginLab | 2022 |
| LivingImage | PerkinElmer | 4.4 |

**Table S6:Other (e.g. drugs, proteins, vectors etc.)**

| **Name** | **Supplier** | **Cat no.** |
| --- | --- | --- |
| Insulin solution human | Sigma-Aldrich | I9278 |
| LY294002 | MedChemExpress | HY-10108 |
| LY303511 | MedChemExpress | HY-15643 |
| Wortmannin | MedChemExpress | HY-10197 |
| PKH26 | MedChemExpress | HY-D1451 |
| Tumor necrosis factor alpha-induced protein 8 | MedChemExpress | HY-P76682 |
| GW4869 | Beyotime | S1971 |
| Cycloheximide | MedChemExpress | HY-12320 |
| Chloroquine | MedChemExpress | HY-17589A |
| MG132 | Sigma-Aldrich | M7449 |
| Gemcitabine | MedChemExpress | HY-17026 |
| Puromycin | Sigma-Aldrich | 540411 |
| Lipofectamine® 3000 | Invitrogen | L3000150 |
| D-Luciferin potassium salt | Macklin | D812647 |
| DiR | MedChemExpress | HY-D1048 |
