# Supplementary material for: An Insulin‐Exosome‐TNFAIP8 Axis Drives Stromal Fibrosis and Therapeutic Resistance in Pancreatic Cancer
Source: Adv Sci (Weinh). 2026 Feb 19;13(24):e15606. doi: 10.1002/advs.202515606 (PMC13116271; doi:10.1002/advs.202515606)
Supplement: Supplementary file 2 — Supporting File 2: advs74468‐sup‐0002‐FigureS1‐S10.docx. [file ADVS-13-e15606-s001.docx]

**
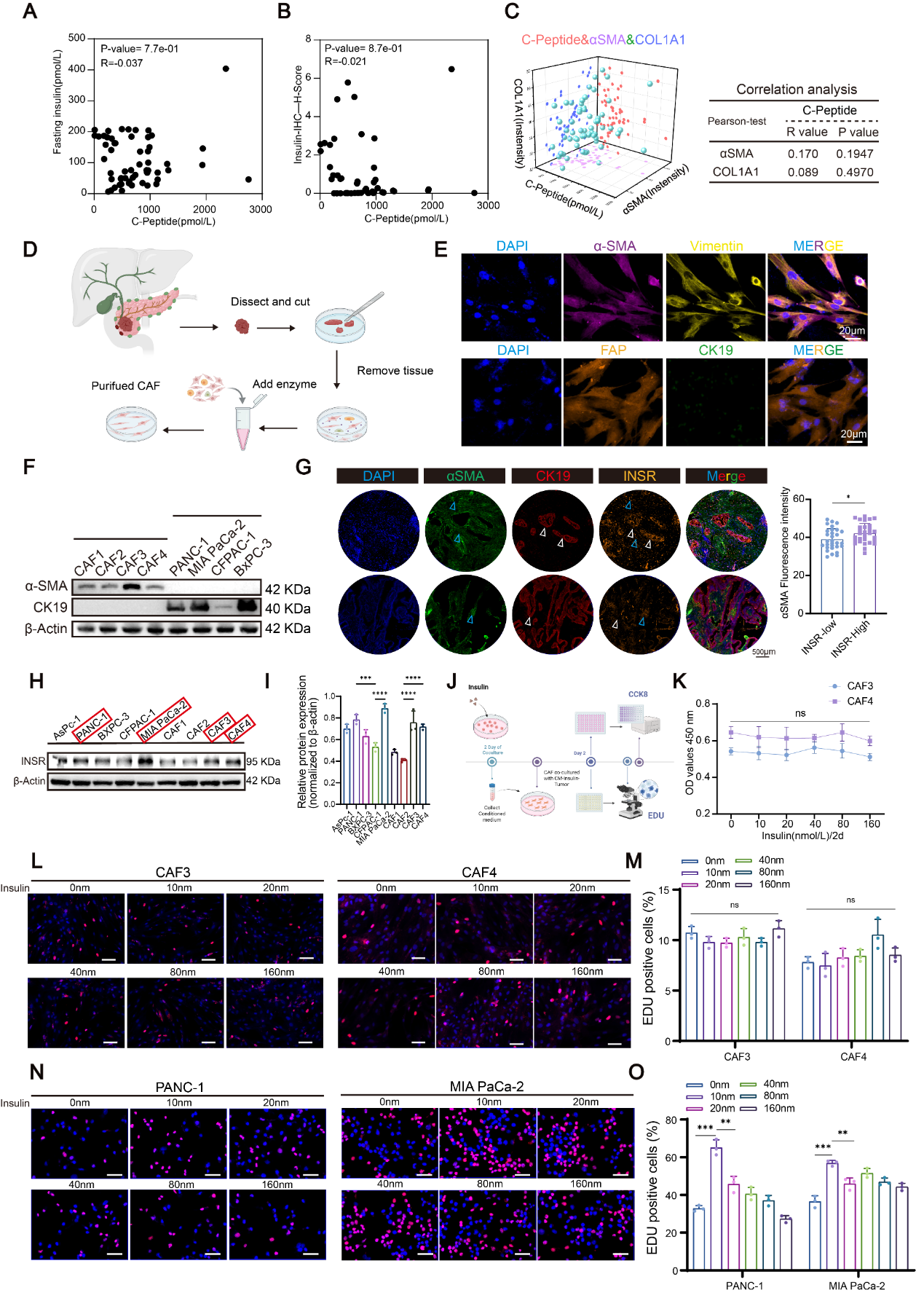
**

**Figure S1. CAF characterization and validation of insulin-responsive models.** (A) Scatter plot showing the relationship between fasting serum insulin levels and C-peptide concentrations in PDAC patients. (B) Scatter plot showing the relationship between tumor insulin IHC H-score and serum C-peptide levels in PDAC patients. (C) 3D quantification correlation analysis of serum C-peptide levels with αSMA and COL1A1 fluorescence intensities in PDAC tissues. Pearson correlation coefficients and P values are indicated. (D) Schematic workflow for isolation and purification of primary CAFs from human PDAC tissues. (E) IF staining showing CAF markers (α-SMA, Vimentin, FAP) and epithelial marker (CK19); scale bars, 20 μm. (F) Western blot analysis of α-SMA and CK19 in primary CAFs (CAF1–CAF4) and PDAC cell lines (PANC-1, MIA PaCa-2, CFPAC-1, BxPC-3). (G) Representative multiplex IF images of PDAC tissue sections stained for DAPI (blue), αSMA (green), CK19 (red), and INSR (orange), with merged images shown on the right. Quantification of αSMA fluorescence intensity in INSR-low and INSR-high tumors is shown. Scale bar, 500 μm. (H) Western blot showing INSR expression in pancreatic cancer cells and CAFs. (I) Western blot analysis of INSR protein expression in PDAC cell lines and CAFs, with densitometric quantification normalized to β-actin shown on the right. (J) Schematic of experimental design: CM from insulin-treated tumor cells applied to CAF cultures for EdU and CCK-8 assays. (K) CCK-8 assay measuring proliferation of CAF3 and CAF4 treated with varying insulin concentrations (0–160 nM) for 2 days (n = 3 biological replicates). (L–M) EdU staining and quantification for CAF3 and CAF4 exposed to different insulin concentrations for 2 days (n = 3 biological replicates). (N-O) EdU assay for PANC-1 and MIA PaCa-2 tumor cells treated with varying insulin concentrations (0–160 nM) (n = 3 biological replicates). Data are presented as mean ± SD and were analyzed by one-way ANOVA with Tukey’s post hoc test (G, I, K, M, O). Correlation analyses were conducted using Pearson’s correlation coefficient (A, B, C, I, J). Significance thresholds: ns, not significant; *P < 0.05, **P < 0.01, ***P < 0.001, ****P < 0.0001.


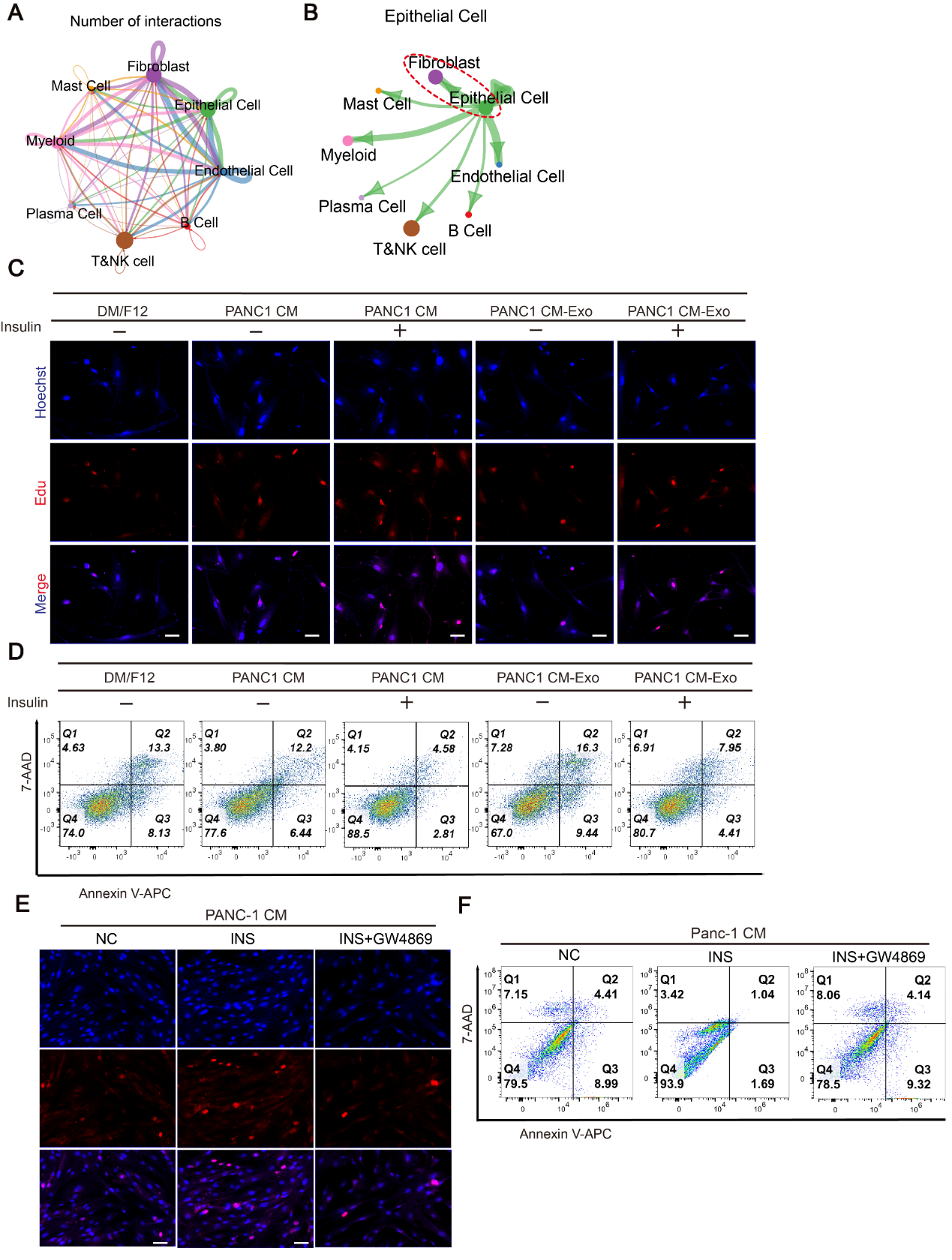
 **Figure S2. Insulin-enhanced epithelial–fibroblast communication promotes CAF proliferation and survival via exosome-dependent mechanisms** (A) Global cell–cell communication network based on inferred ligand–receptor (cytokine/growth factor) interactions among major cell populations. (B) Directional ligand–receptor signaling network centered on epithelial cells. (C) EdU staining of CAF proliferation treated with DMEM/F12, PANC-1 CM, insulin-treated PANC-1 CM, and exosomes isolated from these media (PANC-1 CM-Exo, Insulin PANC-1 CM-Exo). Scale bars, 50 µm. (D) Annexin V/7-AAD assay showing CAF apoptosis rates under different CM and exosome treatments. (E) EdU staining of CAF proliferation treated with PANC-1 CM, insulin-treated PANC-1 CM, insulin+GW4869-treated PANC-1 CM. (F) Annexin V/7-AAD assay showing CAF apoptosis rates under different CM.

**
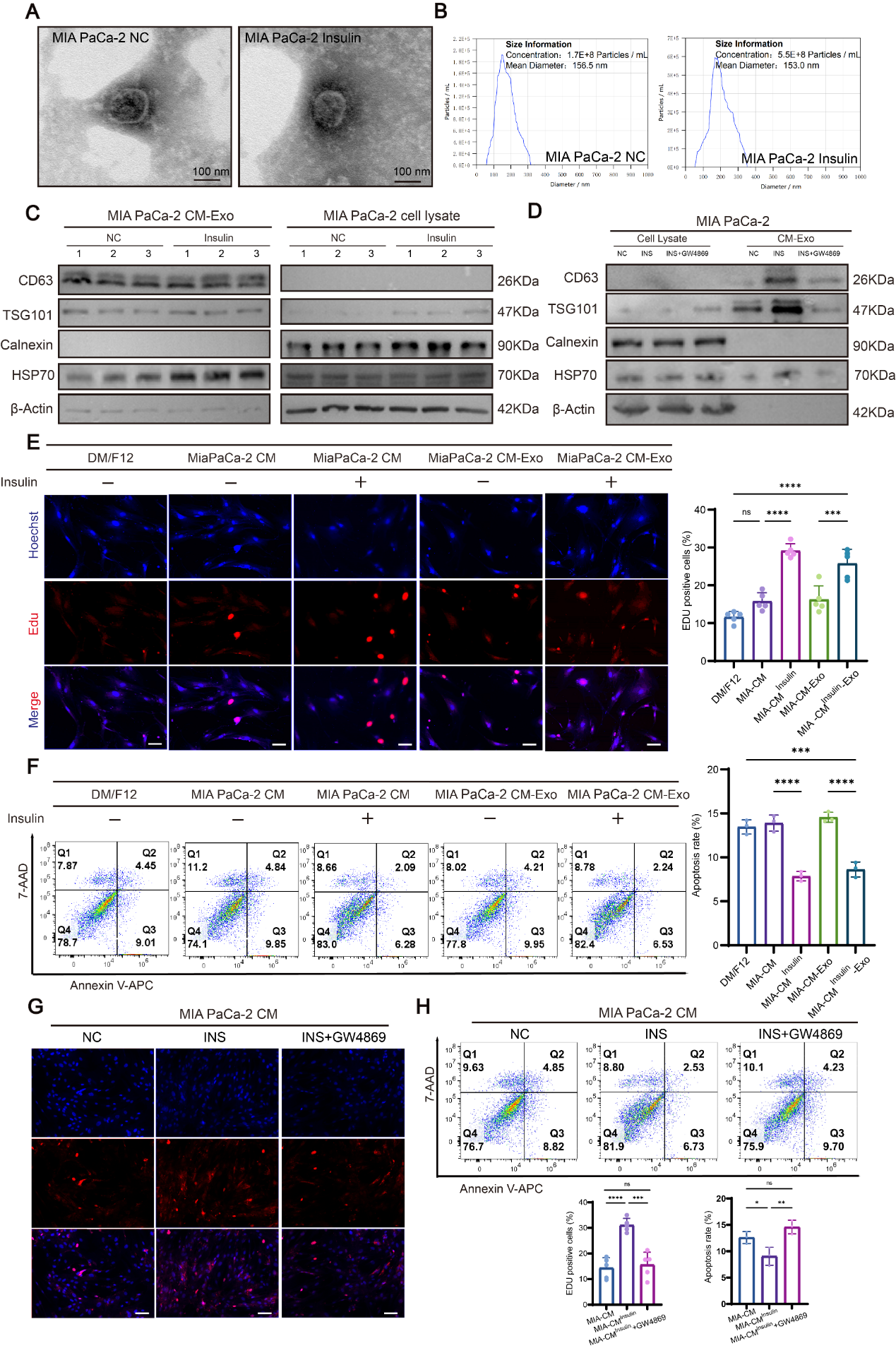
**

**Figure S3. Insulin-driven exosome release from PDAC cells modulates CAF proliferation and apoptosis.** (A) TEM images of exosomes isolated from MIA PaCa-2 conditioned media with or without insulin stimulation. Scale bars,100 nm. (B) NTA of exosome size distribution and concentration from control and insulin-treated MIA PaCa-2 cells. (C) Western blot validation of exosomal markers (CD63, TSG101) and negative control (Calnexin) and HSP70 in exosome fractions and corresponding cell lysates from MIA PaCa-2 cells treated with or without insulin. (D) Western blot analysis confirming the effect of the exosome inhibitor GW4869 on exosomal protein markers in MIA PaCa-2 cells and CM. (E) EdU staining and quantification of CAF proliferation treated with DMEM/F12, MIA PaCa-2 CM, insulin-treated MIA PaCa-2 CM, and exosomes isolated from these media (MIA PaCa-2 CM-Exo, Insulin MIA PaCa-2 CM-Exo) (n = 5 biological replicates). Scale bars, 50 µm. (F) Annexin V/7-AAD assay showing representative plots and quantification of CAF apoptosis under different CM and exosome treatments (n = 3 biological replicates). (G) EdU staining of CAF after treatment with CM from MIA PaCa-2 cells under control, insulin, or insulin plus GW4869 conditions. Scale bars, 50 µm. (H) Representative Annexin V–APC/7-AAD flow cytometry plots showing apoptosis of CAFs treated with MIA PaCa-2–derived CM under the indicated conditions. (I) Quantification of EDU-positive CAFs (n = 5 biological replicates) and apoptotic CAFs (n = 3 biological replicates) corresponding to (G) and (H). All data are presented as mean ± SD and were analyzed by one-way ANOVA with Tukey’s post hoc test (E, F, I). Significance thresholds: ns, not significant; *P < 0.05, **P < 0.01, ***P < 0.001.

**
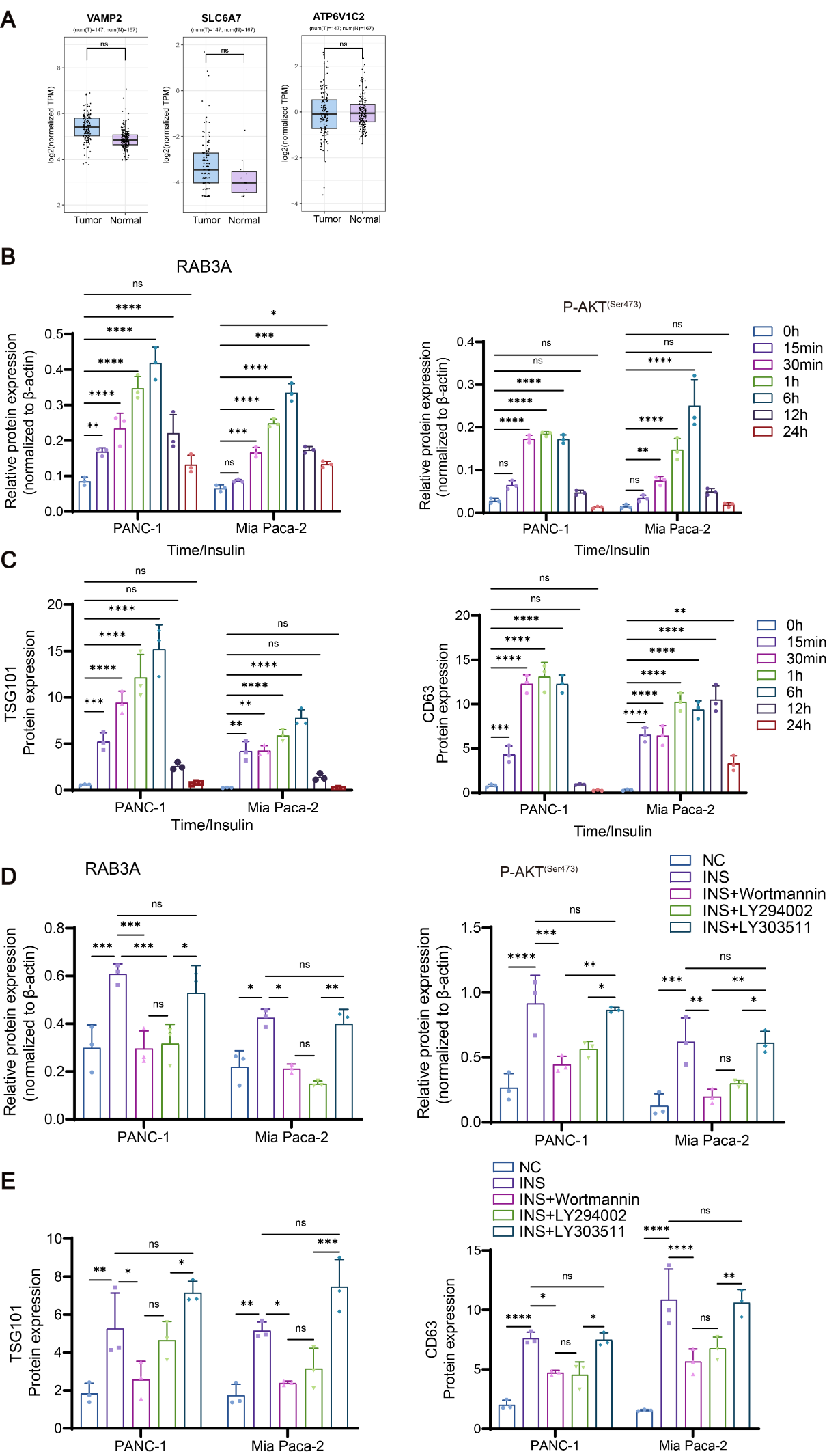
**

**Figure S4. Quantitative Western blot analysis of the insulin–AKT–RAB3A axis** (A) Comparison of synaptic vesicle–related gene expression (VAMP2, SLC6A7, and ATP6V1C2) between tumor and normal tissues based on normalized TCGA PDAC datasets. (B) Time-course quantitative analysis of RAB3A and phosphorylated AKT (Ser473) protein levels in PANC-1 and MIA PaCa-2 cells following insulin stimulation, normalized to β-actin. (C) Quantification of exosome-associated markers TSG101 and CD63 in PANC-1 and MIA PaCa-2 cells at indicated time points after insulin treatment. (D) Effects of PI3K–AKT pathway inhibition (Wortmannin, LY294002, LY303511) on insulin-induced RAB3A and p-AKT (Ser473) expression, as determined by densitometric analysis of Western blots. (E) Quantitative analysis of TSG101 and CD63 expression under PI3K–AKT pathway inhibition, indicating AKT-dependent regulation of exosome biogenesis downstream of insulin signaling. Data are presented as mean ± SEM from at least three independent experiments. Statistical significance was determined as indicated (*P < 0.05; **P < 0.01; ***P < 0.001; ****P < 0.0001; ns, not significant).


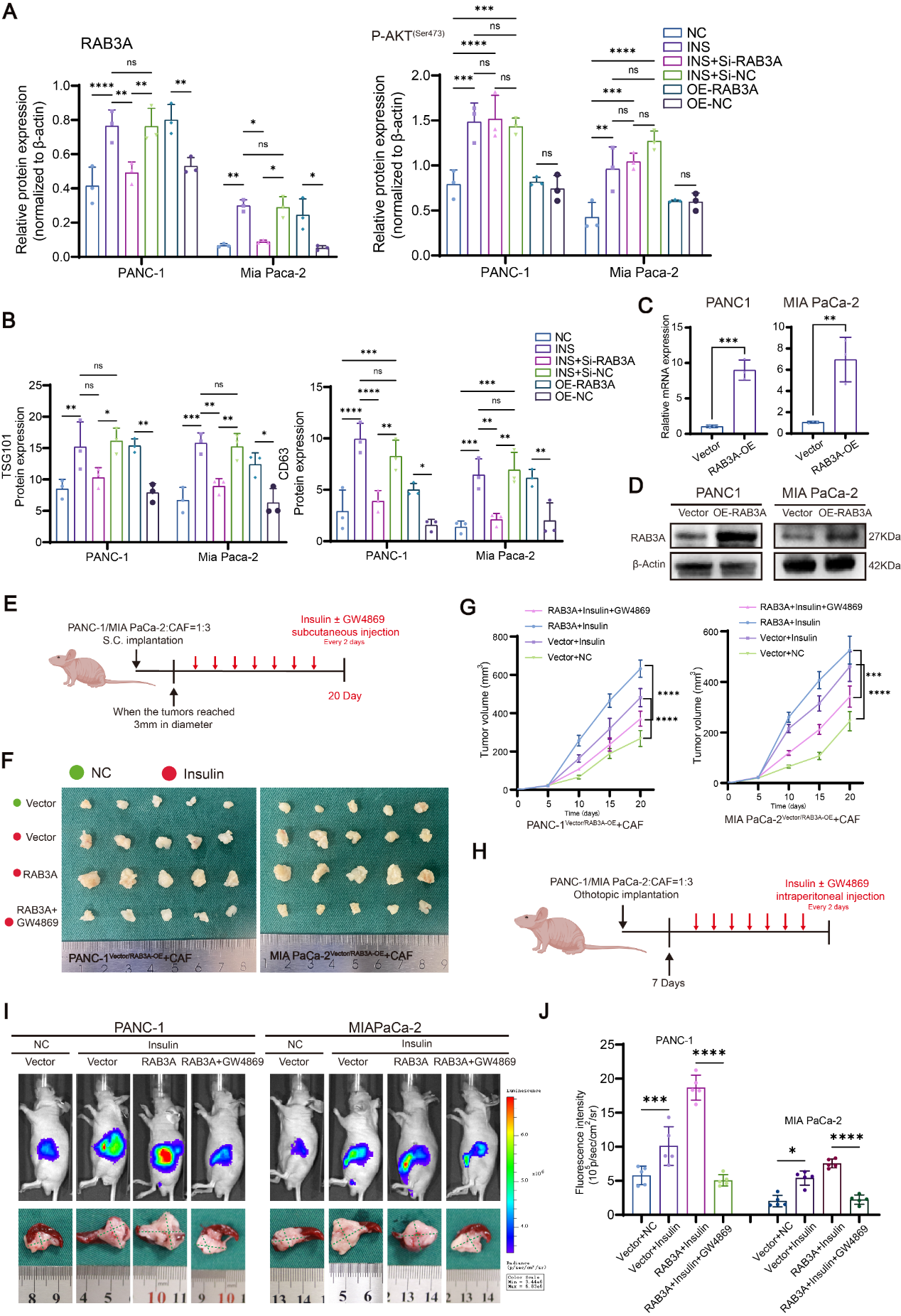


**Figure S5. Insulin promotes RAB3A-mediated tumor growth in vivo, which is inhibited by GW4869.** (A, B) Densitometric quantification of RAB3A, phosphorylated AKT (Ser473), and exosome-related proteins (TSG101 and CD63) in PANC-1 and MIA PaCa-2 cells under insulin stimulation with RAB3A knockdown or overexpression, normalized to β-actin. (C) qPCR analysis of RAB3A mRNA expression in PANC-1 and MIA PaCa-2 cells following RAB3A overexpression. (D) Representative Western blot images showing RAB3A protein levels in PANC-1 and MIA PaCa-2 cells. (E) Schematic diagram of subcutaneous implantation: PANC-1/MIA PaCa-2 cells were mixed with CAFs (ratio 1:3) and implanted subcutaneously; insulin ± GW4869 was administered by subcutaneous injection every 2 days once tumors reached ~3 mm in diameter. (F) Representative subcutaneous tumors from different groups. (G) Tumor growth curves for PANC-1^Vector/RAB3A-OE^+CAF and MIA PaCa-2^Vector/RAB3A-OE^+CAF xenografts. Tumor volumes were measured every 5 days (n = 5 mice per group). (H) Schematic diagram of orthotopic implantation: PANC-1/MIA PaCa-2 cells with CAFs were orthotopically implanted; insulin ± GW4869 was given by intraperitoneal injection every 2 days beginning 7 days post-implantation. (I) Bioluminescence imaging and isolated pancreatic tumor images from orthotopic tumor-bearing mice. (J) Quantification of tumor fluorescence intensity in orthotopic models (n = 5 mice per group). All data are presented as mean ± SD and were analyzed by one-way ANOVA with Tukey’s post hoc test (A, B, C, G, J). Significance thresholds: *P < 0.05, **P < 0.01, ***P < 0.001; ****P < 0.0001.


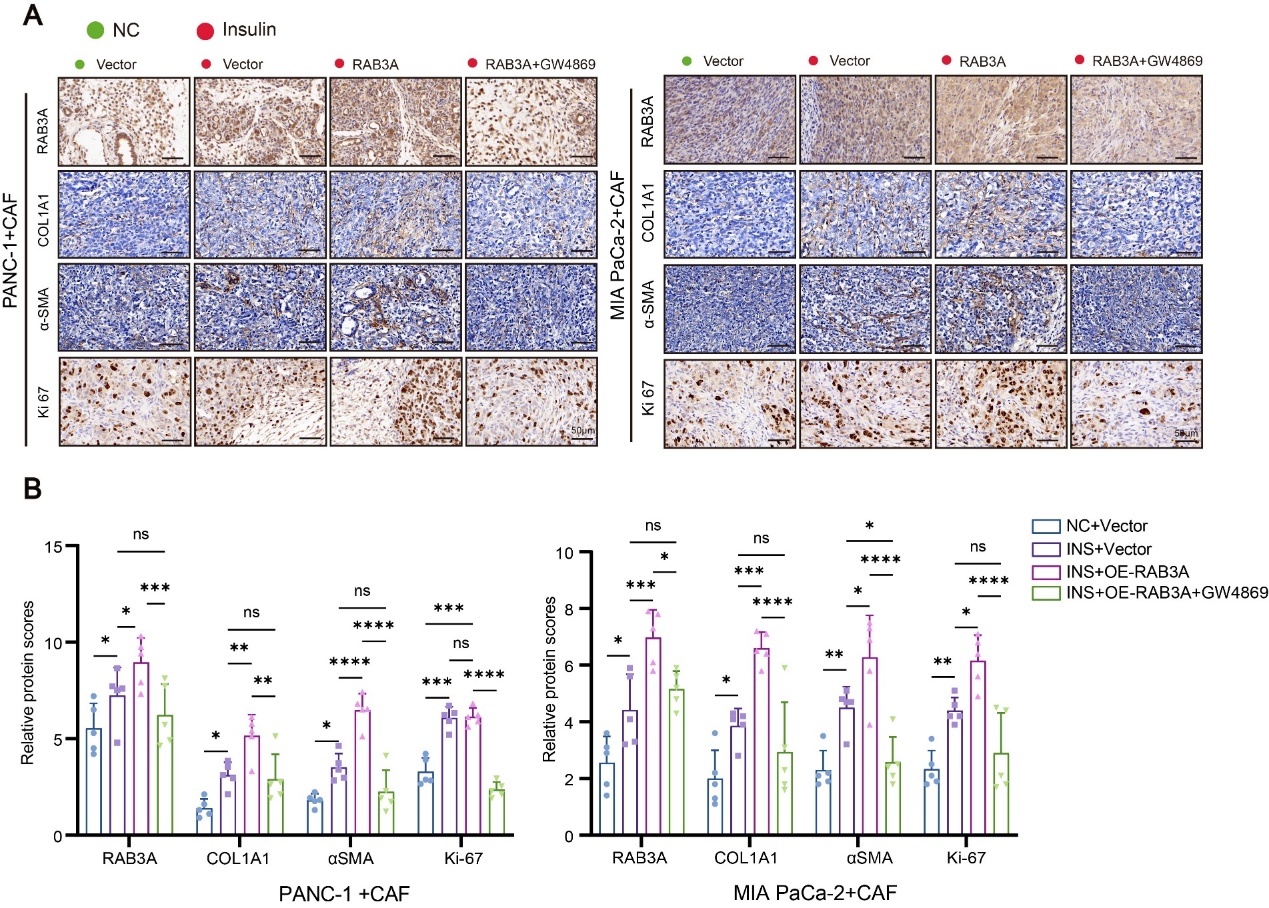


**Figure S6. Insulin promotes RAB3A-mediated tumor growth in vivo, which is inhibited by GW4869.** (A) Representative IHC staining of RAB3A, COL1A1, αSMA, and Ki-67 in orthotopic tumors generated by co-implantation of PANC-1 or MIA PaCa-2 cells with CAFs under the indicated conditions (NC, insulin, RAB3A overexpression, and insulin plus GW4869). Scale bars, 50 μm. (B) Quantification of IHC staining scores for RAB3A, COL1A1, αSMA, and Ki-67 in PANC-1 + CAF and MIA PaCa-2 + CAF tumors. All data are presented as mean ± SD and were analyzed by one-way ANOVA with Tukey’s post hoc test (B). Significance thresholds: ns, not significant; *P < 0.05, **P < 0.01, ***P < 0.001, ****P < 0.0001.


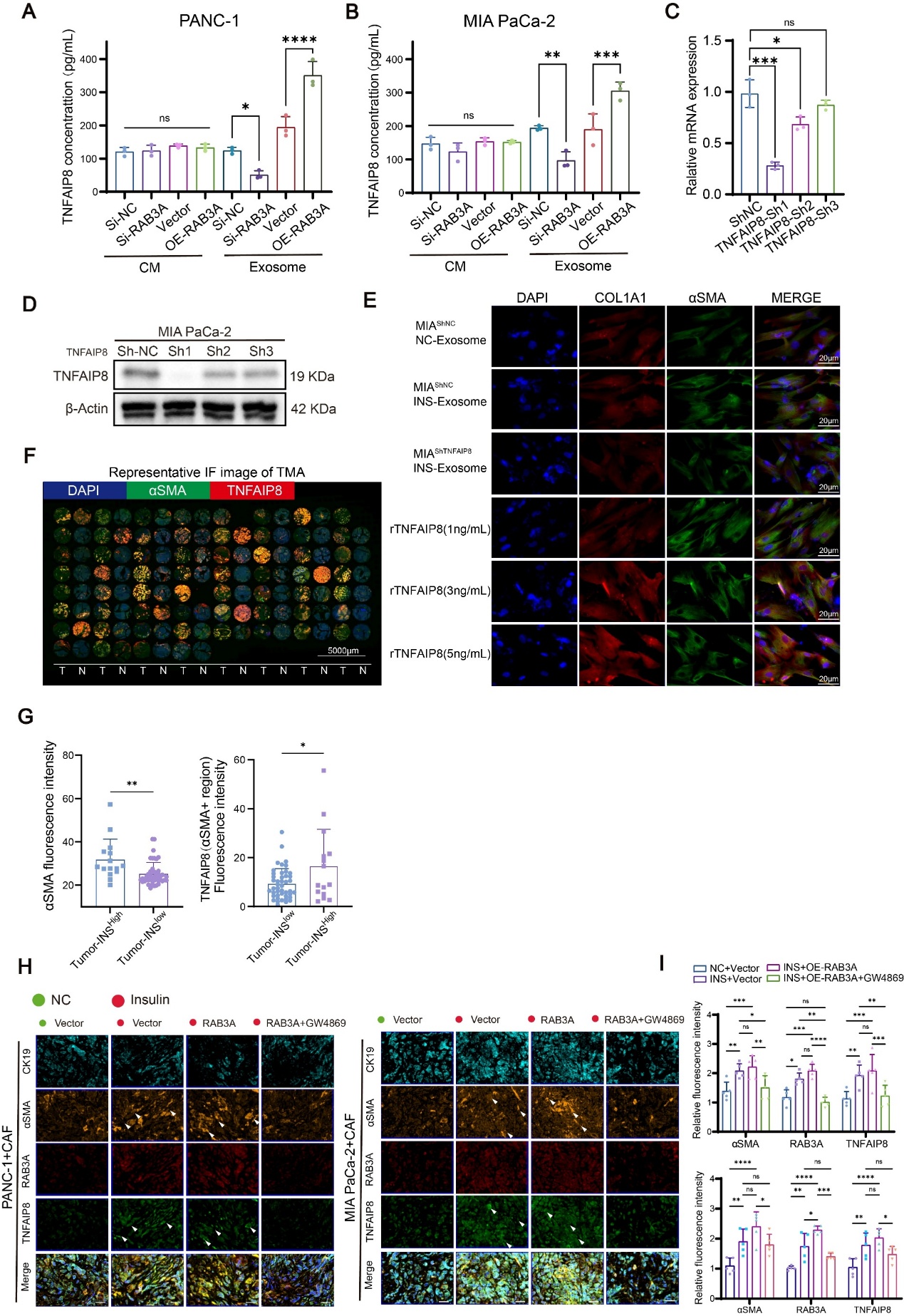


**Figure S7. Exosomes-TNFAIP8 regulate CAF activation and fibrosis-related features in CAFs and PDAC Tissues** (A, B) ELISA quantification of TNFAIP8 concentrations in CM and isolated exosomes from PANC-1 (A) and MIA PaCa-2 (B) cells following RAB3A knockdown or overexpression. (C) qRT–PCR analysis of TNFAIP8 mRNA levels in MIA PaCa-2 cells transfected with control or TNFAIP8 shRNAs. (D) Immunoblot analysis confirming TNFAIP8 knockdown efficiency in MIA PaCa-2 cells. (E) Representative IF images of CAFs treated with indicated exosomes or rTNFAIP8, stained for COL1A1 and αSMA. Scale bars, 20 μm. (F) Representative multiplex IF images of tissue microarrays stained for αSMA and TNFAIP8. Scale bar, 5000 μm. (G) Quantification of αSMA and TNFAIP8(αSMA+ region) fluorescence intensities corresponding to panel F, reflecting stromal fibrosis and TNFAIP8 expression levels. (H) Representative IF images of orthotopic pancreatic tumors in nude mice, generated using PANC-1 or MIA PaCa-2 cells co-implanted with CAFs under indicated treatments. (I) Quantification of αSMA, RAB3A, and TNFAIP8 fluorescence intensities in orthotopic tumor tissues shown in panel H. All data are presented as mean ± SD and were analyzed by one-way ANOVA with Tukey’s post hoc test (A, B, C, I). Statistical comparisons between two groups were performed using a two-tailed unpaired Student’s t-tests (G), Significance thresholds: ns, not significant; *P < 0.05, **P < 0.01, ***P < 0.001, ****P < 0.0001.


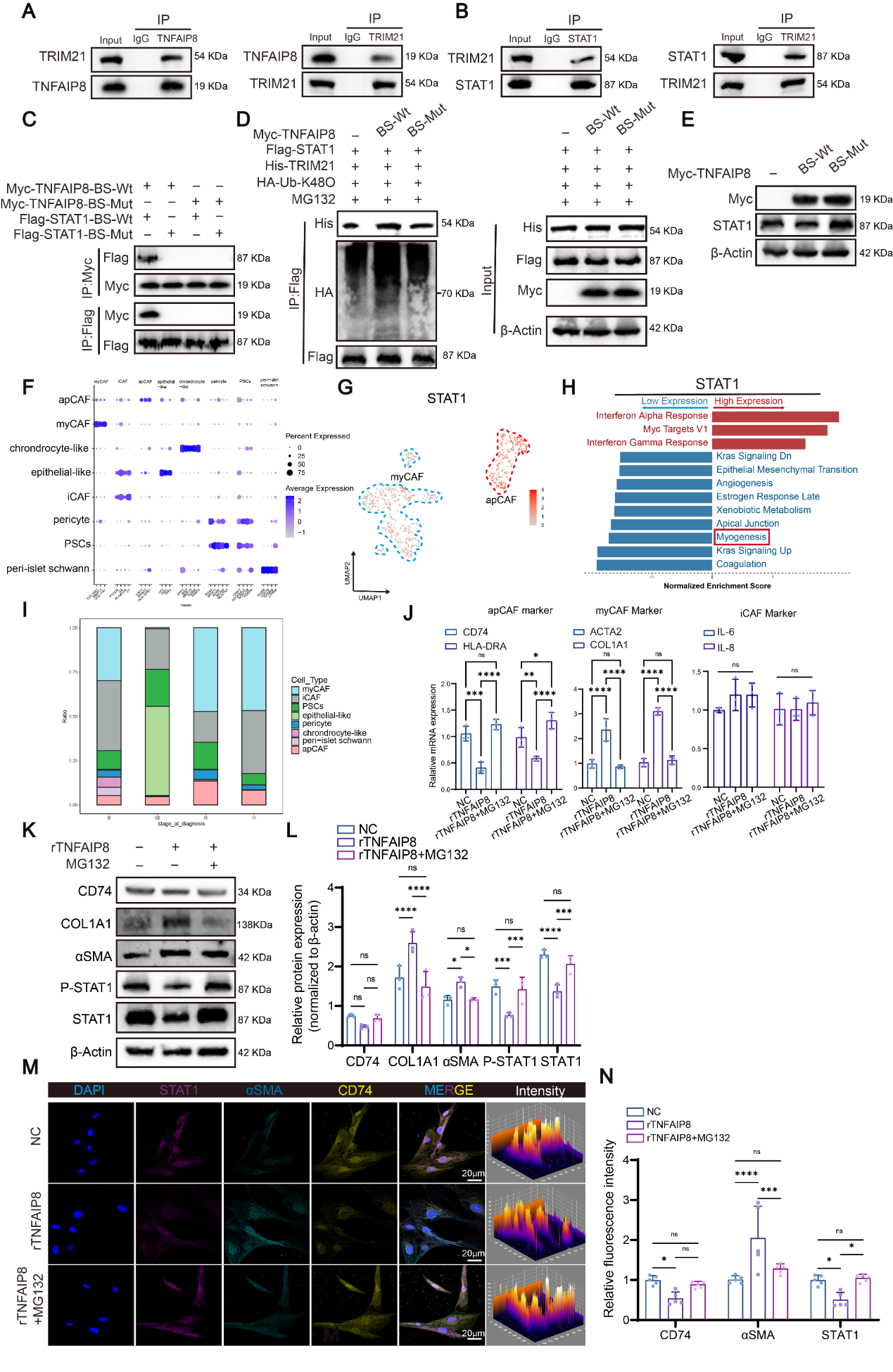


**Figure** **S8. TNFAIP8 interacts with TRIM21 to facilitate STAT1 ubiquitination, supporting CAF subtype–associated phenotypic remodeling.** (A–B) Endogenous Co-IP assays demonstrating interactions among TNFAIP8, TRIM21, and STAT1 in CAF lysates. (C) IP analysis demonstrating the interaction between Myc-TNFAIP8-BS-Wt or Myc-TNFAIP8-BS-Mut and Flag-STAT1-BS-Wt or Flag-STAT1-BS-Mut in HEK293T cells. (D) Assessment of STAT1 ubiquitination levels in HEK293T cells transfected with either Myc-TNFAIP8-BS-Wt or Myc-TNFAIP8-BS-Mut constructs. (E) Western blot analysis of STAT1 protein expression in HEK293T cells following transfection with Myc-TNFAIP8-BS-Wt or Myc-TNFAIP8-BS-Mut. (F) Dot plot showing feature gene expression among CAF subtypes and stromal cells (GSE205013). (G) UMAP plot mapping STAT1 expression in apCAF and myCAF populations. (H) GSEA analysis revealing differential pathway enrichment between STAT1-High and STAT1-Low groups in CAFs. (I) Bar graph showing distribution of CAF subtypes across PDAC stages (IB–IV). (J) QPCR analysis of representative apCAF, myCAF, and iCAF marker genes in CAFs under rTNFAIP8 modulation, with or without MG132 treatment. (K, L) Western blot analysis and corresponding quantification of CAF subtype–associated proteins and STAT1 signaling components following rTNFAIP8 treatment and proteasome inhibition. (M, N) Quantification of CD74, αSMA, and STAT1 IF intensities in CAFs under indicated conditions. All data are presented as mean ± SD and were analyzed by one-way ANOVA with Tukey’s post hoc test (J, L, N). Significance thresholds: ns, not significant; *P < 0.05, **P < 0.01, ***P < 0.001, ****P < 0.0001.

**
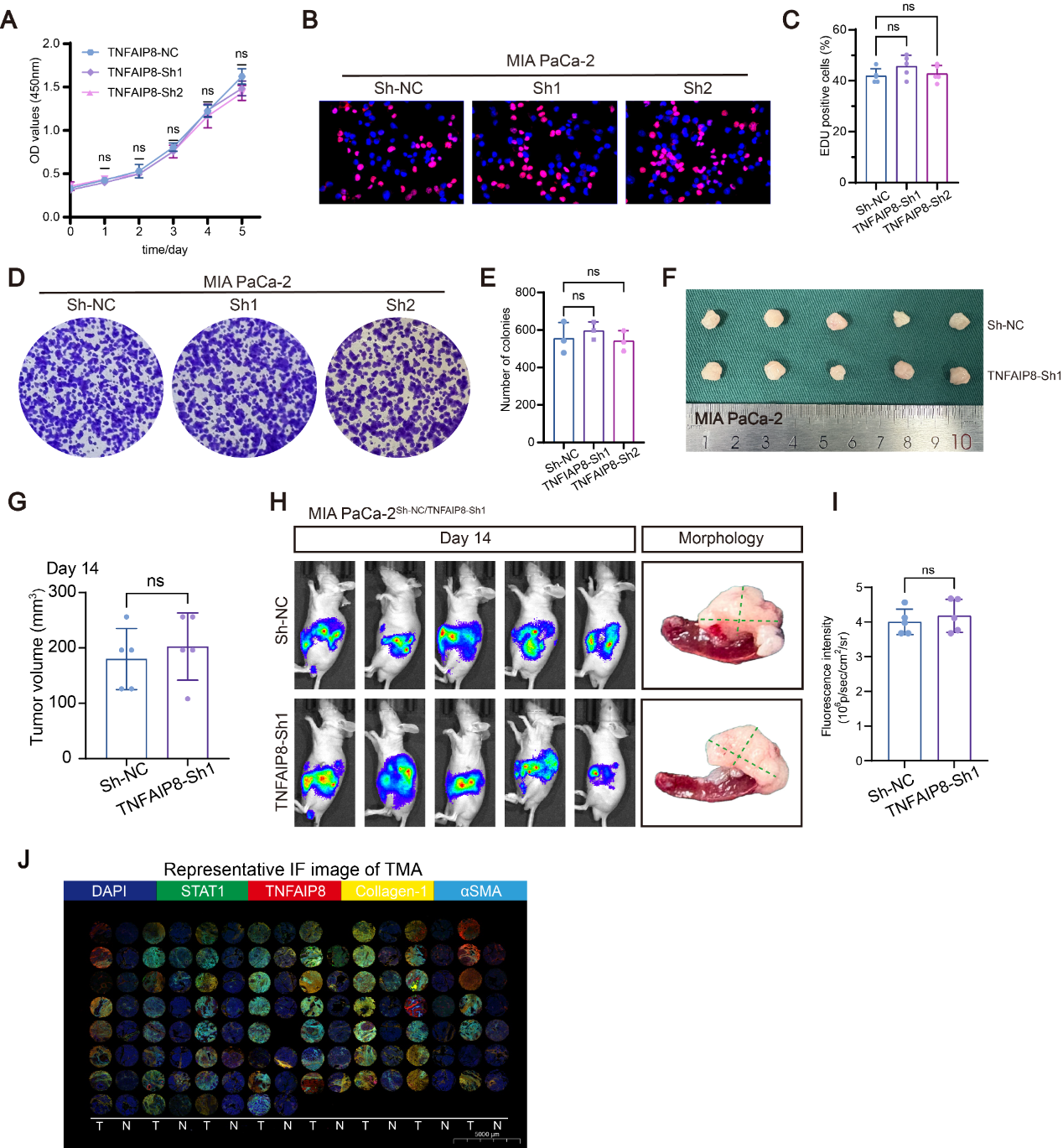
**

**Figure S9. Effects of TNFAIP8 knockdown on PDAC cell growth in vitro and in vivo.** (A) CCK-8 assays showing cell viability of MIA PaCa-2 cells transduced with control shRNA (Sh-NC) or two independent TNFAIP8 shRNAs (Sh1, Sh2). (B) Representative EdU incorporation images of MIA PaCa-2 cells under the indicated conditions. (C) Quantification of EdU-positive cells. (D) Representative colony formation assays of MIA PaCa-2 cells with TNFAIP8 knockdown. (E) Quantification of colony numbers. (F) Representative images of subcutaneous xenograft tumors derived from Sh-NC or TNFAIP8-Sh1 MIA PaCa-2 cells. (G) Tumor volume measurements at day 14 after implantation. (H) Representative in vivo bioluminescence imaging and corresponding gross tumor morphology at day 14. (I) Quantification of bioluminescence signal intensity. (J) Representative multiplex IF staining of PDAC TMA showing nuclei (DAPI, blue), STAT1 (green), TNFAIP8 (red), Collagen-1 (yellow), and α-SMA (cyan) in tumor (T) and adjacent normal (N) tissues. Scale bar, 5000 µm. All data are presented as mean ± SD and were analyzed by one-way ANOVA with Tukey’s post hoc test (A, C, E). Statistical comparisons between two groups were performed using a two-tailed unpaired Student’s t-tests (G, I), Significance thresholds: ns, not significant.


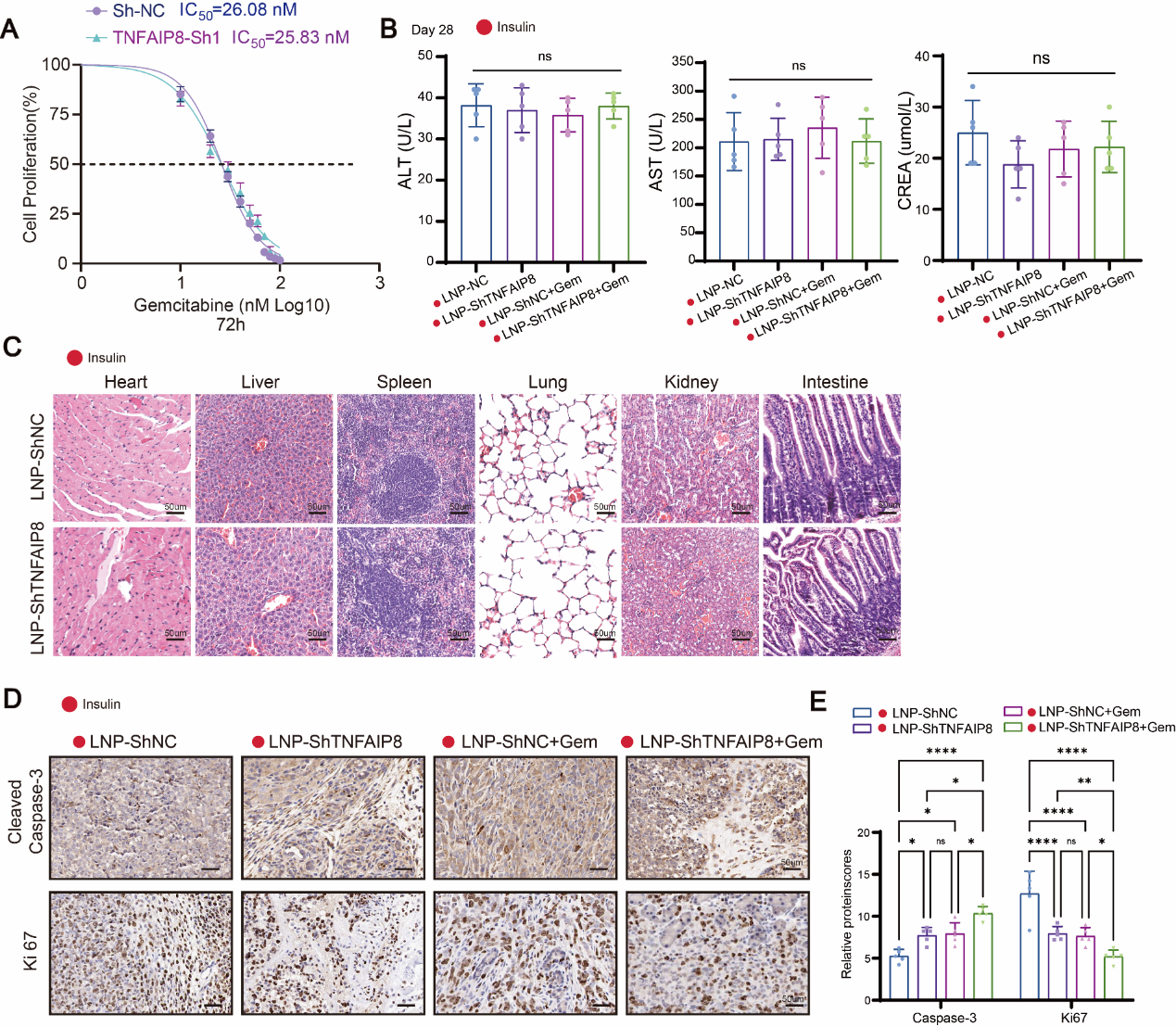


**Figure S10. Assessment of Antitumor Activity and Biosafety of LNP-shTNFAIP8.** (A) Gemcitabine dose–response curves and IC₅₀ determination in MIA PaCa-2 cells expressing Sh-NC or TNFAIP8 shRNA after 72 h treatment. (B) Serum levels of ALT, AST, and CREA measured on Day 28 to assess liver and kidney function (n = 5 mice per group). (C) H&E staining of major organs (heart, liver, spleen, lung, kidney, and intestine) from mice treated with LNP-ShNC or LNP-ShTNFAIP8. Scale bars, 50 μm. (D) Representative IHC staining of cleaved caspase-3 and ki-67 in orthotopic PDAC tumors from mice treated with LNP-ShNC, LNP-ShTNFAIP8, LNP-ShNC+Gem, or LNP-ShTNFAIP8+Gem. Scale bars, 50 μm. (E) Quantification of cleaved Caspase-3 and Ki67 IHC staining in orthotopic PDAC tumors under indicated LNP and gemcitabine treatments (n = 5 mice per group). Dose–response curves and IC₅₀ values were calculated using nonlinear regression analysis (A). Data are presented as mean ± SD and analyzed by one-way ANOVA with Tukey’s post hoc test (B, E). Significance thresholds: ns, not significant.
